# Harnessing the Underutilized Potential of Lens Capsule Transplantation in Ophthalmology: A Narrative Review of Current Applications and Future Directions

**DOI:** 10.7759/cureus.82259

**Published:** 2025-04-14

**Authors:** Joobin Khadamy

**Affiliations:** 1 Ophthalmology, Skellefteå Eye Clinic, Skellefteå, SWE; 2 Ophthalmology, University Hospital of Umeå, Umeå, SWE

**Keywords:** basement membrane, eye bank, glaucoma surgery, lens capsule, macular hole, ocular surface reconstruction, ophthalmic graft, regenerative ophthalmology, tissue engineering, whole eye transplantation

## Abstract

The human lens capsule is a transparent and durable basement membrane routinely discarded during cataract surgery, exhibiting unique biochemical, biomechanical, and immunologic properties. This narrative review discusses validated ophthalmic applications across corneal, retinal, and glaucoma filtration surgeries. It emphasizes the capsule's emerging role as a tissue-engineering scaffold for cultivating corneal endothelial cells, limbal epithelial stem cells, and retinal pigment epithelial cells, demonstrating significant promise in regenerative ophthalmology. However, variability in harvesting techniques, small graft sizes, and limited long-term clinical data currently hinder its broader clinical implementation.

Future directions highlight the necessity of standardizing capsule harvesting and preservation protocols, potentially in collaboration with eye banks, to enhance accessibility and utility. Additionally, this review explores speculative applications, including encapsulation devices for drug and cell delivery, ultraviolet cross-linking for keratoconus management, and novel scaffolds for optic nerve regeneration and retinal transplantation. While preliminary evidence strongly supports the capsule's versatility, rigorous clinical trials and comparative analyses remain essential to establish long-term safety, efficacy, and optimal surgical integration. Ultimately, harnessing this naturally available biomaterial represents a meaningful advancement in ophthalmology, opening new horizons for future research.

## Introduction and background

The lens capsule is the thickest basement membrane in the human body, enveloping the ocular crystalline lens. Composed of interwoven networks of type IV collagen, laminin, nidogen, heparan sulfate proteoglycans, and other matrix components, it provides a uniquely robust yet transparent enclosure for the lens​ [1,2]. Despite its abundance and ready availability as a byproduct of cataract surgery, this biological membrane has historically been overlooked as a surgical resource. Instead, ophthalmic surgeons have favored donor tissues such as amniotic membrane for surface reconstruction, Descemet’s membrane for endothelial grafts, and synthetic implants for various repairs. The lens capsule’s potential has remained underrecognized in comparison, perhaps due to its small size and the traditional focus on intraocular lens (IOL) implantation rather than grafting during cataract surgery.

Growing evidence suggests that the lens capsule can serve as an autologous or allogeneic graft with broad utility across ophthalmic subspecialties. Its biochemical properties resemble those of other basement membranes (including corneal and retinal membranes), yet with a thickness (~10-20 μm centrally in adults) that permits easy handling with microsurgical instruments​ [1]. This is supported by the tolerability of the lens capsule in belt-loop fixation techniques for in-the-bag scleral fixation of IOLs [3], where haptics are looped through capsular remnants to anchor the IOL. Although the capsule is not directly sutured in that context, the fact that surgical manipulation and tension-bearing placement are tolerated suggests that micro-sutures could be applied directly to the capsule when needed.

The capsule is semi-permeable, allowing diffusion of nutrients while filtering intermediate-sized molecules by charge and size [4]. Notably, it shares a similar molecular weight cutoff to Bruch’s membrane in the choroid, hinting at its suitability as a substitute support structure for retinal cells [5]. The immunologic privilege of the lens is well known, the lens epithelium expresses little to no major histocompatibility complex (MHC) class I, and the intact capsule is thought to help maintain the immune seclusion of lens proteins [6]. Immune reactions might still occur depending on graft preparation or cellular remnants; thus, careful decellularization remains crucial. Lens capsules show resistance to enzymatic degradation and infection [7]; they can withstand collagenases from neutrophils and remain uninflamed even as xenografts in short-term studies [8]. These attributes suggest that a transplanted capsule could integrate with minimal immune reaction or fibrosis.

In recent years, innovative surgeons and researchers have begun repurposing the lens capsule in various ocular surgeries. Case reports and small series have described using capsular flaps to close persistent macular holes (MHs) in the retina when internal limiting membrane (ILM) tissue was lacking, with encouraging success [9]. In the cornea, experimental transplantation of an anterior lens capsule has promoted corneal ulcer healing, effectively replacing a damaged Bowman’s layer [10]. Investigators have also used the capsule as a scaffold for cell culture: corneal endothelial cells and limbal stem cells readily grow on this substrate, maintaining phenotype and viability [11]. Furthermore, a prospective trial in glaucoma combined cataract surgery with autologous capsular patch grafts under the scleral flap, significantly improving trabeculectomy outcomes [12]. These disparate applications highlight the lens capsule’s versatility as a transparent, biocompatible membrane that can be transplanted into various ocular compartments.

This narrative review examines the current evidence for using the lens capsule in ophthalmology, spanning its biochemical characteristics, historical and emerging clinical uses, methods for harvesting and preservation, and specific applications in retina, cornea, and glaucoma surgery. We also discuss speculative future uses, from serving as an internal barrier in aphakic eyes with silicone oil to patching defects and aiding ocular surface reconstruction. Challenges and future perspectives are addressed, including the need for standardized preparation techniques and further studies to fully understand long-term outcomes. By compiling and analyzing the literature to date, we aim to shine a light on this underutilized ocular tissue and encourage further research into integrating lens capsule grafts into mainstream ophthalmic practice.

## Review

Methods

A literature search was performed in PubMed to identify studies on lens capsule grafts or transplantation in ophthalmology. The search strategy was: ("lens capsule"[Title/Abstract] OR "lens capsules"[Title/Abstract]) AND ("graft"[Title/Abstract] OR "transplantation"[Title/Abstract]). No date restrictions were applied, and relevant articles were reviewed. Additional sources were obtained by screening reference lists of pertinent papers and including key preclinical and clinical studies on novel uses of the lens capsule. Data on biochemical composition, interspecies comparisons, historical and current clinical applications, and experimental uses of the lens capsule were extracted. Emphasis was placed on preserving details from pivotal studies while synthesizing an original narrative.

Biochemical characteristics

The lens capsule is a smooth, elastic basement membrane chiefly composed of type IV collagen fibrils interlaced with laminin and glycosaminoglycans [1,2]. Ultrastructurally, it resembles other basement membranes in architecture but is dramatically thicker. Human anterior lens capsules average 12-16 μm in thickness in adulthood (increasing with age), compared to the under 5 μm thickness of Descemet’s membrane or the ILM. Posterior capsule regions are thinner (~4 μm centrally), but even these rival the thickness of many biologic membranes [13,14]. This substantial thickness contributes to its mechanical strength and ease of handling during surgery [15]. Anterior capsule thickness facilitates easier surgical manipulation, making it preferable for structural grafts, whereas posterior capsules may be ideal for more delicate procedures due to their thinner profile.

The capsule’s matrix composition is remarkably conserved across species [13,16]; analyses in mammals (including primates, pigs, and rabbits) have found similar profiles of collagen IV, entactin/nidogen, heparan sulfate proteoglycans (with perlecan being predominant), and collagen XVIII (an anti-angiogenic molecule) [1,17]. Minor differences exist, for example, capsular thickness can vary (porcine anterior capsules are about 8-10 μm, somewhat thinner than human), but these differences do not significantly alter its functional properties as a scaffold [17]. Cross-species transplantation studies have shown that even xenogeneic lens capsules can be tolerated short-term in the eye without inciting severe inflammation [8], underscoring the matrix’s low immunogenicity when decellularized. While minor, these variations may influence mechanical handling, cell adhesion properties, or permeability, necessitating careful evaluation before clinical translation of xenografts.

Biochemically, the capsule behaves as a molecular sieve. It is permeable to water and small nutrients required for lens metabolism, while selectively impeding larger molecules based on size or charge [4]. Its diffusion characteristics parallel those of Bruch’s membrane in the retina-choroid interface, with a similar cutoff (~100-150 kDa), which has invited comparisons and experiments using the capsule as an in vivo surrogate for Bruch’s membrane. The capsule’s laminin-rich inner surface naturally supports adhesion of epithelial cells (lens epithelium in situ), suggesting that other cell types (corneal epithelium, endothelium, retinal cells) can likewise attach firmly to the capsule’s basement membrane-like matrix [4,5,18-21]. Unlike synthetic membranes, the lens capsule also contains bioactive cues; for instance, collagen XVIII can generate endostatin, which may confer anti-angiogenic properties to suppress unwanted neovascularization in transplanted settings [1,22,23].

Notably, the lens capsule contributes to the immune-privileged status of the lens. The intact capsule sequesters lens proteins from the immune system, and lens epithelial cells behind it express negligible MHC-I [24,25]. This means an autologous lens capsule graft is essentially non-immunogenic, and even an allograft may be tolerated if cellular antigens are removed. In experimental lens capsule transplants across individuals and species, minimal inflammatory responses have been observed [24,26,27]. Additionally, the capsule shows resistance to common ocular pathogens and proteolytic enzymes. Studies have demonstrated that lens capsules are not readily degraded by neutrophil elastase or bacterial collagenases [28,29]. This inherent resistance to enzymatic lysis, perhaps due to densely crosslinked collagen IV, bodes well for its stability as a graft in inflamed or infected environments [29,30]. In summary, the lens capsule’s conserved biochemical makeup and unique permeability profile enable it to serve as a biologically friendly scaffold, and these properties hold true across various species and contexts (Table 1). This has encouraged the use of animal lens capsules in some research as stand-ins for human tissue, with promising translational implications.

**Table 1:** Characteristics and potential ophthalmic applications of the lens capsule. ILM: Internal limiting membrane; RPE: Retinal pigment epithelium; CCC: Continuous curvilinear capsulorhexis; UV: Ultraviolet.

Property	Description and Implication for Ophthalmic Use
Thickness Variation	Anterior capsule: thicker (8-15 μm), suitable for structural patches; Posterior capsule: thinner (~4 μm), delicate scaffolds comparable to internal limiting membrane (ILM) or Descemet’s membrane.
Transparency	High clarity, minimal impact on optical performance; ideal for visual-axis grafts such as corneal and macular applications.
Biomechanical Strength	High tensile strength and elasticity; suitable for tension-bearing grafts (e.g., glaucoma bleb reinforcement, corneal tectonic support).
Permeability (Molecular)	Semi-permeable basement membrane (~100-150 kDa cutoff), allows nutrient diffusion; similar to Bruch’s membrane, suitable for subretinal scaffolding.
Immune Privilege	Extremely low immunogenicity (especially autologous); can be transplanted without rejection or significant inflammation.
Resistance to Degradation	High resistance to proteolytic enzymes (collagenases); theoretically ideal in infected or inflamed environments; potential for ocular surface reconstruction.
Cell Adhesion & Integration	Supports epithelial, endothelial, RPE, and limbal stem cell adhesion; proven compatibility as a scaffold for cultured cell transplantation.
Cross-Linking Potential (UV)	Speculative use of UV cross-linking for controlled stiffening; potential application in corneal structural reinforcement (e.g., keratoconus).
Encapsulation Potential	Speculative; potential to encapsulate therapeutic agents or cells (e.g., insulin-producing cells, stem cells) for drug or cell delivery.
Antimicrobial Potential	Theoretical resistance to macrophage- or neutrophil-mediated degradation; resistance to fungal/bacterial colonization is speculated but requires confirmation.
Clinical & Surgical Handling	Excellent handling characteristics: minimal curling, robust for suturing, easily manipulated intraoperatively, suitable for microsurgical procedures.
Harvesting & Preservation	Easily harvested intraoperatively (continuous curvilinear capsulorhexis (CCC), push-pull technique, femtosecond laser, or Zepto precision pulse capsulotomy (PPC)); potential for standardized eye bank preservation (cryopreservation, lyophilization).

Historical and emerging clinical use

Historical Use

The concept of using the lens capsule as a surgical graft dates back several decades in experimental settings. One of the earliest applications was in retinal surgery: researchers in the 1990s explored the capsule as a scaffold for retinal pigment epithelium (RPE) transplantation in models of macular degeneration [31,32]. Hartmann et al. reported that human and porcine anterior lens capsules could support the growth and grafting of RPE and iris pigment epithelium (IPE) cells in vitro [32]. This suggested that a lens capsule might replace Bruch’s membrane as a foundation for new RPE cells. Building on this idea, Kiilgaard and colleagues performed subretinal implantation of allogenic anterior lens capsules in pigs, aiming to substitute for damaged Bruch’s membrane [33]. They found the grafted capsules were well-tolerated and, within days, became covered by a monolayer of host RPE, provided the native Bruch’s membrane remained intact [33]. These pioneering studies from 1999-2002 demonstrated the capsule’s biocompatibility in the subretinal environment and its ability to encourage retinal cells to migrate and adhere. Around the same time, ocular surface researchers in Eastern Europe applied lens capsules in corneal ulcer repair. Kozák et al. transplanted anterior lens capsules onto the corneas of rabbits with chronic epithelial ulcers as a replacement for the missing Bowman’s layer [10]. The outcomes showed that the lens capsule grafts integrated into the corneal stroma and supported the re-epithelialization of persistent ulcers, effectively acting like an “artificial Bowman’s layer” to stabilize the corneal surface. An ultrastructural follow-up study confirmed that these allografted capsules remained in place and that regenerating corneal epithelium developed normal adhesion complexes with the capsule beneath [34].

In the 2000s, corneal tissue engineering efforts further expanded the lens capsule’s role. Galal et al. in Spain published a seminal study using human anterior lens capsules as a substrate for cultivating limbal stem cells ex vivo [11,35,36]. Limbal biopsies grown on the capsule achieved a confluent epithelial cell sheet with 95% viability and morphology comparable to controls. This 2007 work established proof of concept that a patient’s own lens capsule (obtained during cataract surgery) could be repurposed to grow autologous corneal epithelium for treating limbal stem cell deficiency [11]. Likewise, Szurman and colleagues used anterior lens capsules to culture corneal endothelial cells, positing the capsule as a carrier for endothelial grafts [37]. By 2009, they demonstrated that human endothelial cells formed a monolayer on the capsule with pump function proteins (Na⁺/K⁺ ATPase, ZO-1 tight junctions) appropriately expressed, a critical step toward a tissue-engineered endothelial keratoplasty graft. These early clinical and laboratory investigations, spanning retina to cornea, laid the groundwork by highlighting the capsule’s compatibility with various ocular cells and its integration in living eye tissues.

Despite promising early results, widespread adoption was hindered by limited clinical awareness, complexity in graft handling, and the absence of standardized methods for harvesting and preservation.

Emerging Use

In the last decade, interest in the lens capsule has resurged, driven by practical surgical needs and innovations. One breakthrough came in vitreoretinal surgery for MHs. Chronic or large MHs sometimes cannot be closed by standard ILM peel and gas tamponade, especially if the ILM has been exhausted from prior surgery. In 2016, Chen and Yang reported an alternative: using an autologous lens capsular flap to bridge the MH [38]. In their series, a free anterior capsule flap (harvested during phacoemulsification) was placed into the MH, leading to hole closure in 75% of refractory cases. This innovative technique quickly gained traction. Peng et al. combined the lens capsular flap with autologous whole blood as an adhesive in seven phakic and three aphakic eyes, achieving 90% complete closure of persistent MHs and significant visual improvement [39]. Subsequent reports have corroborated these successes. Cisiecki et al. described five cases of large, chronic MHs that were all successfully closed with autologous anterior lens capsule transplantation, with an average preoperative hole size of ~667 µm [9]. Remarkably, even complex scenarios like multiple concurrent MHs have been managed with this technique: one case report detailed the repair of a retinal detachment caused by multiple eccentric MHs using a tailored capsular flap to cover all the holes, resulting in retinal reattachment and visual improvement [40]. These emerging retinal applications mark the lens capsule’s entry into mainstream vitreoretinal practice as a readily available graft for otherwise intractable cases. However, current evidence predominantly relies on small, uncontrolled series and case reports; larger, randomized controlled trials are necessary to draw definitive conclusions about efficacy and safety.

In glaucoma surgery, the lens capsule’s emerging use is as an anti-fibrotic patch to enhance bleb function. Initial observations came from Lu et al. in 2009, who noted improved outcomes when placing an anterior lens capsule under the scleral flap during combined phacoemulsification-trabeculectomy [12]. A more definitive study in 2016 by Das et al. involved 88 patients randomized to phacotrabeculectomy with or without an autologous capsular graft [41]. The results at three months were striking: 93.2% of eyes with a capsular implant achieved complete success (IOP ≤21 mmHg without medications) compared to 70.5% without the implant. Mean IOP was significantly lower in the capsule-augmented group (11.1 vs. 17.4 mmHg) at all follow-ups. Bleb morphology grading showed fewer encapsulated (scarred) blebs and more diffusely functioning blebs when the capsule was used. No increased complication was observed; in fact, no cases of bleb leak or infection were noted in the capsule group. These findings have positioned the lens capsule as a potential natural substitute for mitomycin-C or other anti-scarring measures in glaucoma filtration surgery. While not yet widely adopted, this emerging evidence indicates that a humble piece of the patient’s own lens capsule can markedly improve surgical success in glaucoma by acting as a spacer and modulating wound healing [12].

Finally, in corneal surgery, clinical translation has been more gradual but is now materializing. The concept of using a lens capsule as a corneal patch graft remained largely experimental until very recently. In 2023, Murthy et al. evaluated anterior lens capsule (ALC) transplantation for corneal perforations [42]. They harvested ALC grafts from donor eyes undergoing femtosecond laser-assisted cataract surgery (FLACS), folded them twice, and positioned these grafts directly over the corneal defect. The grafts were secured using cyanoacrylate glue followed by the placement of a bandage contact lens (BCL). The outcomes demonstrated enhanced epithelial healing and improved patient comfort, suggesting promising clinical utility.

Expanding on these developments, in 2025, Kymionis et al. reported the first human case of using an allogeneic anterior lens capsule transplant to treat a persistent neurotrophic corneal ulcer [43]. A donor capsule obtained from cataract surgery was placed over a non-healing herpetic ulcer as a biological bandage. The capsule effectively acted as a scaffold for epithelialization, achieving corneal surface healing when conventional treatments had failed (as described in the case report). This success in a real-world clinical scenario mirrors earlier animal studies and opens the door for lens capsule use in corneal surface diseases. Another novel frontier is in keratoconus management: in 2024, Rodríguez-Barrientos and colleagues introduced an intrastromal lens capsule graft as a substitute for Bowman layer transplantation in advanced keratoconus [8]. In this pilot study, an isolated anterior lens capsule was implanted into a mid-stromal pocket of a keratoconic cornea, analogous to how donor Bowman layer patches are used to stabilize ectasia. Early results showed the capsule graft integrated as a thin reinforcing layer, improving corneal curvature and thickness over one month post-op [8]. While still experimental, this signals a creative new use case aimed at overcoming donor tissue shortages for Bowman layer grafts.

In summary, what began as scattered experimental uses of the lens capsule has evolved into credible clinical techniques in the 2010s and 2020s. From the retina to the cornea and glaucoma, the lens capsule is shedding its status as “surgical waste” to become a valuable adjunct. The following sections delve into the practical aspects of harvesting and preserving the capsule, detail specific clinical applications with outcomes, and propose future directions building on this momentum.

Harvesting, preservation, and banking

The successful use of lens capsules in surgery depends on proper harvesting and preservation techniques to ensure a clean, intact graft. Unlike amniotic membrane (which is procured from tissue banks), lens capsules are typically harvested directly by the surgeon or eye bank technician.

Harvesting Methods

The most common source is patients undergoing cataract surgery. During phacoemulsification, a continuous curvilinear capsulorhexis (CCC) of about 5 mm in diameter is created in the anterior lens capsule. This transparent circular disc can be carefully retrieved instead of being discarded. Anterior capsule buttons obtained in this manner have been used as autologous grafts in MH surgery and glaucoma procedures [9,38,39]. Alternative capsulorhexis methods, such as the push-pull technique, may offer the advantage of yielding an intact, undistorted circular ALC, which is particularly valuable when a defect-free graft is required for transplantation, as these methods do not necessitate a central punch. To maximize the available graft size, femtosecond laser-assisted capsulotomy has been suggested, as it can create a precise, larger capsular disc (up to 5.5 mm or more) with strong continuous edges [44-46]. A recent report utilizing FLACS-derived capsules for corneal ulcer repair exemplifies the potential for donor-derived capsule use. However, that study did not clarify whether grafts were used immediately or stored, and omitted critical steps such as sterility assurance, decellularization, or media selection, highlighting the need for standardized reporting protocols [42].

In cases where a larger patch is needed or the patient is pseudophakic or aphakic, donor eye globes can serve as sources. Eye banks can isolate the lens by opening the globe, performing a 360° capsular incision at the equator, and peeling off the entire capsule-bag from the lens nucleus. This yields a much larger membrane (approximately 9-10 mm across), comprising anterior and posterior capsule together. Such whole lens capsule bags have been collected for research use in corneal endothelial scaffolds [47]. Posterior capsules alone are thinner and have been less commonly harvested unless the anterior is unavailable, one example being a pseudophakic eye where the intact posterior capsule from a prior cataract surgery could be obtained via a vitrectomy approach, though this is rare [38,48]. Although posterior capsules are thinner and more fragile, they may be preferable in cases requiring minimal scaffold thickness, such as subretinal or ILM-substituting applications. In summary, surgeons can obtain autologous anterior or posterior capsules intraoperatively, and eye banks can harvest larger capsules from donor lenses to provide tissue for allograft use. Xenografts from other species can integrate well into host tissues, as seen in the case of porcine anterior lens capsules used in corneal transplantation. However, the biocompatibility and long-term outcomes of such xenografts need to be thoroughly evaluated [8].

**Figure 1:**
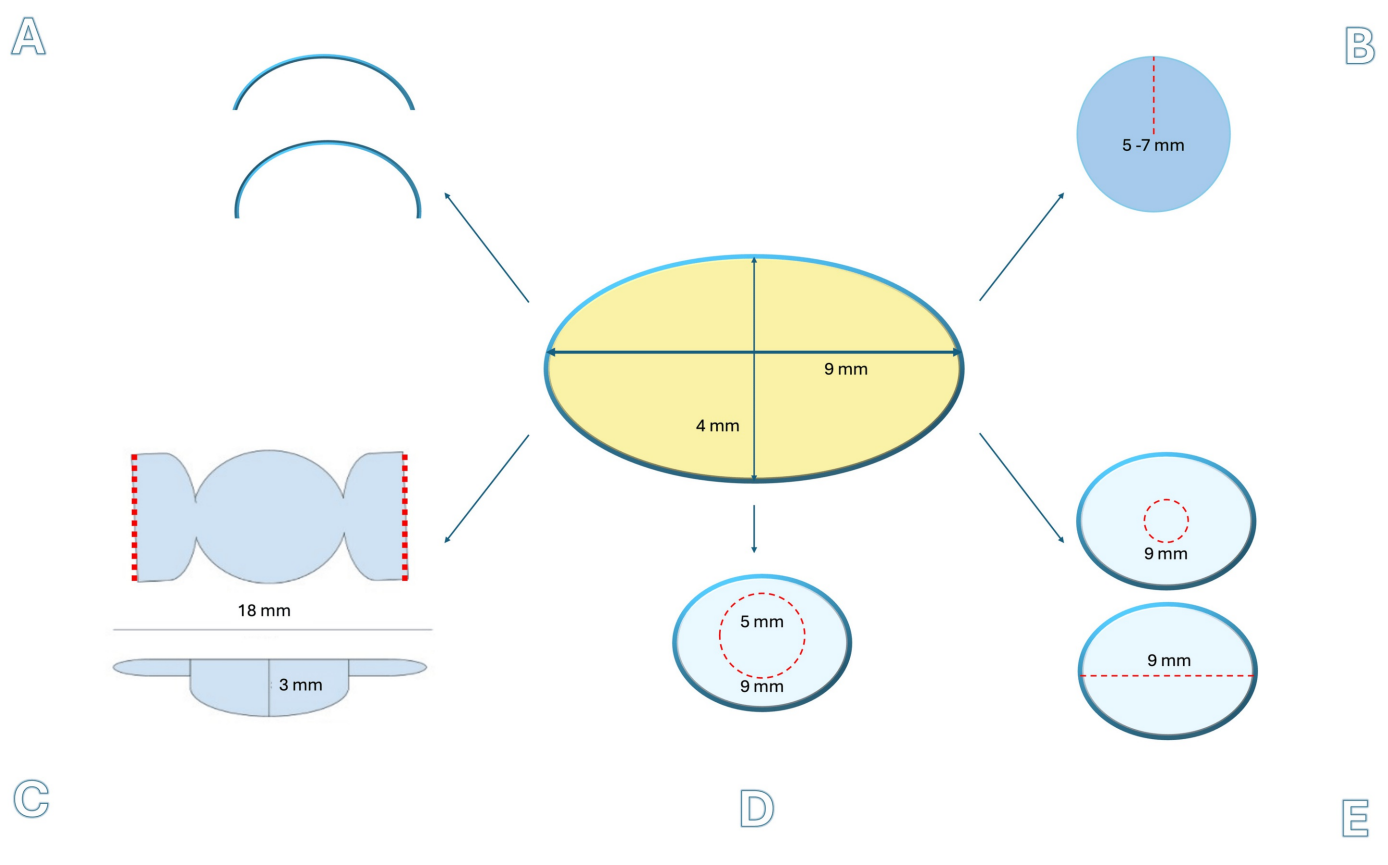
Schematic overview of potential lens capsule harvesting: geometrical and dimensional variations. This schematic outlines four approaches for harvesting and configuring the human lens capsule (~9 mm diameter, 4 mm height) for grafting and tissue engineering. A: Lenticular Arcs: Curved anterior/posterior segments harvested by bisecting the lens at its equator. Different base curvatures suit anatomical conformity (e.g., corneal surface). Speculative; requires scaffolding and biomechanical evaluation. B: Circular Capsule Buttons: 5-7 mm discs harvested via continuous curvilinear capsulorhexis, femtosecond laser, or Zepto nano-pulse. Clinically used for macular hole and optic pit repairs, laser methods improve precision and reproducibility. C: Candy Wrapper Flaps: Speculative method involving a central linear incision and partial equatorial cuts, preserving lateral hinges for folding. This design could serve ocular surface reconstruction, akin to amniotic membrane transplantation. D: Capsular Bag Graft for Aphakia: A centrally capsulorhexised donor capsular bag, mimicking a pseudophakic eye configuration. Used in whole-bag transplantation techniques for secondary IOL implantation in eyes with aphakia. E: En Bloc Capsular Pouch/Reservoir: Entire capsular bag harvested with a central or equatorial opening (upper) for lens aspiration in soft lenses, or via a 150°-180° equatorial incision (lower). Potential uses include drug delivery reservoirs, glaucoma micro-shunt reservoir covers, or cell transplantation. Requires further study. These harvesting strategies vary in clinical maturity. While some are used in practice (e.g., B and D), others (A, C, D) represent speculative or early-stage innovations requiring dedicated instrumentation, safety validation, and application-specific feasibility trials. Together, they reflect the underexplored potential of the lens capsule as a versatile, autologous, and species-cross-compatible ocular biomaterial. Image credit: This figure was originally created by the author for this article and has not been previously published elsewhere.

To better visualize the diversity of harvesting approaches and the potential geometric configurations of the lens capsule, a schematic overview is provided in Figure 1, illustrating both clinically used and speculative designs adapted for various ophthalmic applications.

Decellularization of the Lens Capsule

After excision, effective removal of lens epithelial cells (LECs) from the capsule is essential to prevent postoperative proliferation, fibrosis, or secondary membrane formation. Several methods exist for denuding the capsule of its epithelium, ranging from mechanical techniques to chemical and enzymatic approaches, each with distinct advantages and limitations.

Mechanical scrubbing:Mechanical decellularization is often performed intraoperatively using a spatula, forceps, or silicone-tipped applicator. Surgeons gently scrape the inner (concave) surface of the capsule to dislodge the LECs under an operating microscope, a technique described by Lu et al. and widely adopted in cataract surgery [12]. Nicolini et al. used silicone-coated tips to clean porcine and bovine capsules [49]. However, mechanical removal alone may leave cellular remnants, raising concerns about incomplete decellularization.

Osmotic lysis - distilled water and hypertonic saline:Crowston et al. showed that brief exposure (2 minutes) to sterile distilled water causes osmotic shock and effective LEC lysis without compromising the structural integrity of the capsule [50]. Galal et al. reported complete LEC elimination after a 2-hour soak in deionized water. A 2025 comparative study confirmed that both sterile water (2-4 minutes) and 10% sodium chloride (NaCl) at 37 °C are highly effective at achieving acellularity while preserving capsular collagen and structure [51]. Hypertonic saline not only lyses cells but also appears to preserve hydroxyproline content, suggesting less ECM degradation.

Alcohol-based decellularization: Rodríguez-Barrientos et al. used 70% ethanol to sterilize and decellularize porcine capsules in their keratoconus xenograft study [8]. Ethanol induces protein precipitation and cell lysis while offering surface disinfection. Rinsing in balanced salt solution (BSS) post-treatment is critical to eliminate residual alcohol, which may be cytotoxic.

Hydrogen peroxide (H₂O₂):Although not widely reported in lens capsule preparation, hydrogen peroxide has been used in cataract surgery to suppress posterior capsule opacification. In vitro studies demonstrated that brief exposure to 3% H₂O₂ reduced LEC regrowth on human capsule surfaces [52]. Similar to contact lens disinfection systems, peroxide-treated capsules must be thoroughly rinsed or neutralized before implantation to avoid oxidative damage to host tissue.

Enzymatic digestion:Trypsin and other enzymes offer controlled, reproducible decellularization. Hartmann et al. used 0.05% trypsin on human and porcine capsules for 30 minutes, achieving thorough LEC removal with preserved collagen integrity [32]. Other enzymes like Dispase and TrypLE™ have also been used in research contexts. These methods are less commonly used intraoperatively due to time and rinsing requirements but are well-suited for tissue banking and preoperative preparation.

For sterilization beyond disinfection**:** If the capsule is to be stored or used as an allograft/xenograft, sterility is paramount. Ethanol, povidone-iodine, and antibiotic soaks (e.g., gentamicin solution) have all been used. UV irradiation of the capsule is another option, Hartmann et al. sterilized their capsules under UV light after processing [32]. Gamma irradiation could be a method for sterilizing batch-prepared capsules by eye banks, though it might affect biomechanical properties.

After processing, especially if chemicals like alcohol or peroxide are used, thorough rinsing and soaking in a balanced solution is required to avoid toxicity. Typically, capsules are given multiple washes in sterile BSS. Some protocols add an antibiotic-antifungal soak (like corneal tissue storage media) to ensure no microbial survival.

Finally, verifying that the capsule is indeed acellular can be done by simple microscopy or staining (e.g., trypan blue and alizarin red staining to check for live cells, as used by Yu et al. 2025) [51]. A truly decellularized lens capsule will have no viable cells and minimal cell nuclei on histology (H&E). Ensuring this state is important to prevent any risk of graft-versus-host response or fibrous opacification from residual lens cells (which could proliferate and cause secondary membrane formation, analogous to PCO).

To sum up, the field is converging on reliable methods to produce sterile, acellular lens capsule grafts. For intraoperative use, two of the simplest and most effective are brief exposure to distilled water or hypertonic saline [51]. These require nothing more exotic than what is already present in an ophthalmic OR or lab. For laboratory or banking applications, enzymatic or alcohol-based methods may provide more complete decellularization. In all cases, ensuring acellularity helps minimize fibrosis, immune reaction, and graft failure. Choosing an appropriate method depends on the clinical context. With such protocols in place, it becomes feasible for surgeons around the world to prepare a patient’s own lens capsule during cataract surgery for immediate therapeutic use, or for tissue banks to prepare donor capsules for on-demand use, much like they supply amniotic membrane today. Standardization and training will be key, for example, teaching cataract surgeons how to handle the capsule atraumatically if it is to be repurposed, or instructing retina specialists in the art of placing a transparent graft they’re not used to seeing. Encouragingly, early adopters have published step-by-step techniques for these procedures, which will accelerate broader adoption.

For handling, the capsule’s natural tendency is to curl (with the epithelial side inward) once free from the lens [47]. Surgeons often stain the capsule with a vital dye (trypan blue at 0.06% is commonly used) to enhance visibility [53], and then keep it moist in BSS so it remains pliable. The thin membrane can be draped flat on a surgical sponge or glide for cutting into desired shapes, for example, trimming to just cover a MH or fashioning a strip for a scleral patch. In vitreoretinal cases, 23-25 gauge forceps are used to grasp and insert the capsule flap [39,48], whereas in anterior segment uses, tying or suturing the capsule to host tissue may be employed. Some glaucoma surgeons tuck it under the scleral flap without sutures, secured by normal flap closure [12,41].

*Preservation and Banking* 

Fresh autologous use is the norm, many reports describe harvesting the capsule and immediately transplanting it during the same surgery (e.g., during a combined phaco-vitrectomy for a MH or phaco-trabeculectomy) [9,41]. In such cases, the capsule is simply stored in BSS on the surgical field for minutes to hours until needed. However, for wider adoption, methods to preserve capsules for later use are under exploration. Cryopreservation techniques analogous to amniotic membrane banking have been proposed: a capsule could be placed in glycerol or a cryoprotectant medium and frozen at -80 °C or in liquid nitrogen. A frozen capsule, once thawed, retains its structural integrity and biochemical composition, although some loss of biomechanical strength is possible (this has not been extensively studied yet) [54-59]. Hartmann’s group found that simple freezing with glycerol not only killed lens cells but also helped loosen them for removal [32]. This method could allow eye banks to stockpile capsules from donor eyes (which otherwise go unused if the cornea isn’t harvested) and have them available for surgeons.

Another approach is lyophilization (freeze-drying), which would allow dry storage at room temperature and easy transport; the capsule can be rehydrated in saline before use [60]. Amniotic membranes are sometimes lyophilized for similar reasons. While this has not yet been demonstrated for lens capsules, given their composition, they likely would tolerate freeze-drying well. Care must be taken to ensure no cracking or undue stiffness occurs after rehydration.

These preservation strategies are still largely theoretical, but the feasibility is high given the acellular, collagenous nature of the tissue (similar to dehydrated amnion products).

To date, no eye bank has initiated trial programs to collect anterior lens capsules from donor eyes or surgical centers. Standardization is needed for such “capsule banks” [61]. Key considerations include ensuring sterility (harvesting in sterile conditions, antibiotic soaks), verifying that the tissue is disease-free (screening donors for infectious diseases such as HIV and hepatitis, similar to corneal donation protocols), and documenting capsule size and quality. If these hurdles are overcome, surgeons could gain access to off-the-shelf lens capsule grafts for emergency or elective use, similar to how scleral patch grafts or pericardial grafts are currently supplied. The small size of each capsule is a limitation, but pooling multiple capsules (e.g., quilting or overlapping them for larger corneal surface coverage) is conceivable. The use of animal-derived capsules (e.g., from pig lenses, obtainable from abattoirs) has also been contemplated to supply larger grafts, given the close matrix similarity across species [8,10]. Of course, xenografts would require rigorous evaluation for long-term rejection, but short-term studies in rabbits have not shown immune reactions [8].

**Figure 2:**
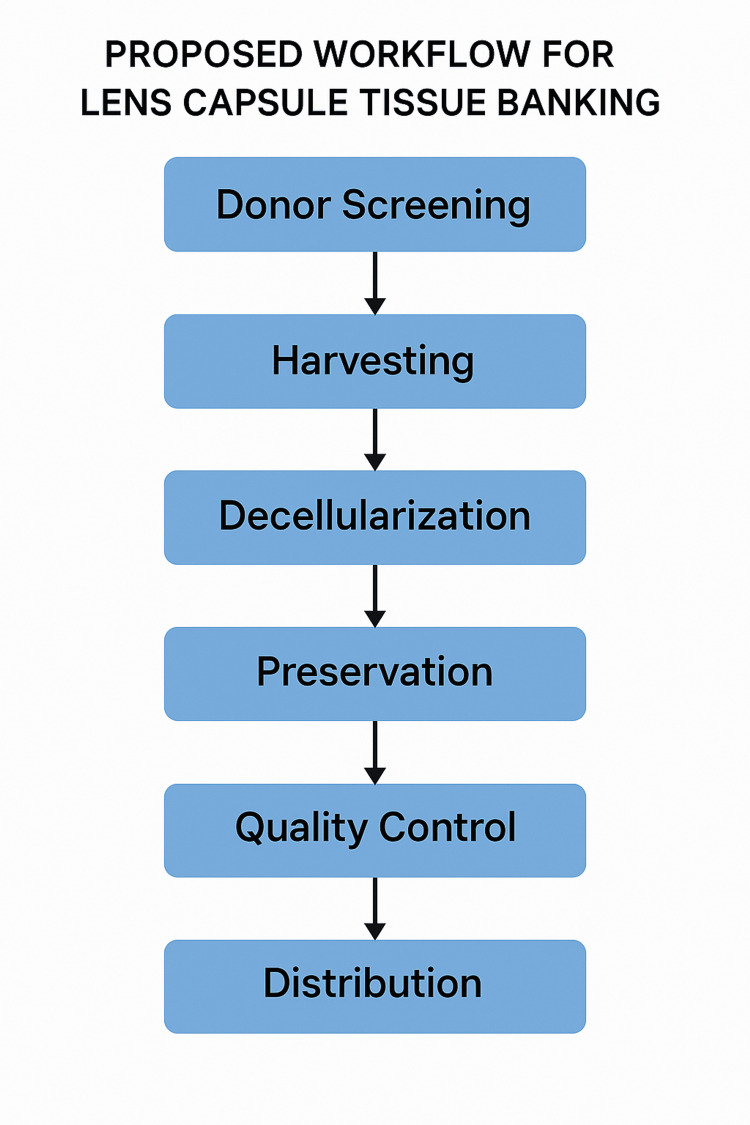
Proposed workflow for lens capsule tissue banking. The process begins with donor screening, followed by harvesting of the capsule, decellularization to remove residual cells, preservation (via cryopreservation or lyophilization), quality control checks, and final distribution to surgical centers. This modular framework parallels current practices in amniotic membrane and corneal tissue banking. Image credit: This figure was originally created by the author for this article and has not been previously published elsewhere.

A proposed tissue banking workflow, encompassing donor screening, harvesting, decellularization, preservation, quality control, and distribution, is illustrated in Figure 2 to facilitate standardization of lens capsule graft preparation.

In summary, lens capsule grafts can be harvested autologously during surgery or obtained from donors, decellularized by mechanical or chemical means, and used fresh or potentially banked for future use. Table 2 encapsulates these methods. Establishing reliable preservation protocols is a key step toward broader clinical availability of this versatile material. The establishment of capsule banks will require compliance with national tissue banking regulations, including sterility, donor consent, and infectious disease screening protocols, akin to current amniotic membrane banking systems.

**Table 2:** Harvesting, decellularization, handling, and preservation methods for lens capsule grafts. In this article, short-term refers to storage for ≤7 days at 4 °C, while long-term preservation refers to cryopreservation or lyophilization exceeding one month.

Stage	Method	Details and Notes
Harvesting	Manual capsulorhexis (CCC)	Continuous curvilinear capsulorhexis (CCC); standard manual technique during cataract surgery yielding 5-6 mm anterior capsule discs. Common in autologous grafting.
Harvesting	Femtosecond laser capsulotomy	Laser-assisted capsulotomy using femtosecond technology; provides smoother, circular edges. Promising for standardizing graft size and shape.
Harvesting	Donor whole lens dissection	The entire capsule bag (anterior and posterior) is removed post-mortem. Suitable for eye bank or research applications.
Decellularization	Mechanical scraping	A spatula or silicone tip is used under a microscope to manually remove lens epithelial cells (LECs). Simple and fast, but may be incomplete.
Decellularization	Distilled water lysis	Osmotic shock using sterile distilled water lyses LECs within 2-4 minutes. Maintains extracellular matrix (ECM) integrity.
Decellularization	Hypertonic saline (10%)	Exposure to 10% sodium chloride causes cellular dehydration and death in 2 minutes. Preserves collagen content; requires precise handling.
Decellularization	Ethanol (70%)	Ethanol denatures cellular proteins and sterilizes the capsule. Requires thorough rinsing to avoid cytotoxicity.
Decellularization	Hydrogen peroxide (3%)	Oxidizes and lyses cells; shown effective in vitro. Must be neutralized or rinsed to prevent toxicity to host tissue.
Decellularization	Trypsin (0.05%)	Enzymatic digestion using trypsin for 30 minutes. Effective in removing LECs while preserving capsule structure. Suitable for laboratory use.
Immediate Handling	Vital dye staining & sizing	Trypan blue (0.06%) enhances capsule visibility; the capsule is trimmed and oriented under a microscope for surgical placement.
Immediate Handling	Surgical placement	Capsule is used as an anterior (e.g., scleral patch) or posterior (e.g., macular hole) graft using microsurgical forceps and fluid/gas tamponade, with or without a blood clot.
Preservation	Fresh (immediate use)	Most reports describe fresh autologous grafts used in the same surgical session. Temporarily stored in balanced salt solution (BSS).
Preservation	Cold storage (short-term)	Short-term preservation at 4°C in sterile media (e.g., Dulbecco’s or Optisol GS). Used for eye bank donor grafts.
Preservation	Cryopreservation	Long-term storage using cryoprotectants (e.g., glycerol) at -80°C. Comparable to amniotic membrane banking. Currently experimental.
Preservation	Lyophilization (drying)	Freeze-drying or air drying of decellularized capsule enables ambient storage. Requires rehydration before use. Investigational stage.
Tissue Banking	Lens capsule tissue banks	Proposal to establish banks for collecting capsular discs from cataract surgeries. Requires donor screening, decellularization, and quality control (QC).

Clinical applications

MH and Optic Disc Pit Maculopathy

Full-thickness MHs are classically treated by pars plana vitrectomy with ILM peeling and gas tamponade, which achieves closure in most cases. However, in chronic, large, or recurrent MHs, standard techniques can fail, especially if the ILM has been removed in a prior surgery. The lens capsule has now emerged as a valuable graft to address these refractory MHs. The principle is analogous to an ILM free flap technique: a piece of transparent basement membrane is placed as a scaffold bridging the retinal gap, inducing glial migration and closure. Chen and Yang’s 2016 report first popularized this approach, using a capsular flap in 20 eyes with stubborn MH from various etiologies [38]. They achieved anatomic closure in 15 of 20 eyes (75%), with corresponding visual improvements in many cases. Since then, several studies have refined the technique. Typically, during the repeat vitrectomy, if the patient is phakic, a cataract extraction is performed and the anterior capsule is obtained via capsulorhexis. In pseudophakic or aphakic patients, a small anterior capsule can be taken from the fellow eye (combined with cataract surgery), or even the posterior capsule remnant if present [9,39]. The capsule flap (often about 4-5 mm) is then transplanted, grasped with ILM forceps, fed through the trocar, and gently laid over or nudged into the MH under fluid. It tends to stay in place due to surface tension and possibly fibrin, but surgeons commonly perform a fluid-air exchange, sometimes with a few drops of whole blood to help “glue” the flap [39]. Gas tamponade (SF₆ or C₃F₈) and face-down positioning for about one week are used to secure the graft.

Outcomes with capsular flap MH repair have been very promising. In Peng et al.’s 2018 series combining autologous lens capsular flap transplantation (LCFT) with autologous blood, 9 out of 10 eyes achieved complete hole closure (the one partially closed case had an extremely large hole) [39]. The median visual acuity improved from 20/1750 pre-op to 20/450 post-op [39]. Cisiecki et al. (2021) reported five persistent MH cases (median minimum diameter ~667 µm), all successfully closed after capsular flap transplantation, with appreciable gains in vision [9]. Notably, the lens capsule’s thicker nature makes it easier to manipulate than an ultra-thin ILM flap, it doesn’t fold over itself as readily and can be directed into position more predictably [9,39]. Some surgeons have observed that an anterior capsule flap, being heavier and less likely to float, stays at the retinal surface more reliably [39]. This can be advantageous in large holes where ILM flaps tend to roll up or dislodge. Compared to ILM flaps, capsule flaps offer greater rigidity, easier manipulation, and may integrate better in large or chronic holes due to their thickness and scaffold effect.

Beyond idiopathic MH, lens capsule flaps have been used in challenging scenarios such as myopic MHs with retinal detachment and multiple eccentric MHs. In one case of retinal detachment with multiple secondary MHs (caused by iatrogenic trauma from prior epiretinal membrane peeling), a single autologous anterior capsule graft was spread to cover several small holes in the macula under perfluorocarbon liquid. After gas tamponade, the retina was reattached and all the holes remained sealed at follow-up, with some visual recovery [40]. This demonstrates the adaptability of the capsule flap, it can conform to irregular or multiple defects in the thin foveal tissue. Another report described using two separate capsular flaps to simultaneously close a reopened central MH and a new adjacent eccentric hole in the same eye, with anatomical success [62].

While the majority of cases have favorable outcomes, the use of lens capsules in the macula is not without caveats. One potential complication is the development of epiretinal proliferation or gliosis on the graft. Temkar et al. (2024) reported a case in which a large MH was successfully closed with an ALC flap, but within a month the patient developed a dense glial plaque over the fovea, causing visual acuity to plateau at 20/200 [53]. In that case, the capsule had not been decellularized (the native LECs were left intact), which likely contributed to fibrocellular proliferation on the retinal surface. This underscores the importance of thorough capsule preparation, residual cells on the capsule can behave like epiretinal membrane cells, causing scar tissue. Fortunately, such reports are rare, and careful techniques (water lysis or peeling off LECs) should mitigate the risk. Additionally, long-term data on visual quality after capsular flap MH surgery are still limited. Although OCT imaging often shows the hole closed with the graft acting as a scaffold, the overlying neurosensory retina may remain thinner or disrupted. In most cases, the graft remains visible on OCT for several weeks post-op but may become less distinct over time. Persistent hyperreflectivity may correlate with gliosis or graft contraction [9,53]. Patients can achieve reading vision, but subtle metamorphopsia may persist depending on photoreceptor realignment [53].

Although anatomic closure is common following lens capsule grafting for MHs, functional restoration of vision may remain suboptimal in some cases, likely due to delayed photoreceptor realignment, gliosis, or persistent disruption of the outer retinal layers. To address this, future strategies could explore combining lens capsule scaffolds with adjunctive therapies such as autologous retinal cell suspensions or stem cell-derived photoreceptor precursors, which may enhance retinal regeneration and improve visual outcomes.

In summary, lens capsule transplantation has become an effective solution for refractory MHs, especially when ILM tissue is unavailable. It boasts high anatomical success rates, can be done autologously in a single surgery, and appears safe with proper preparation. The technique is now a part of the surgical armamentarium for vitreoretinal specialists dealing with complex MHs.

Moving to the optic nerve head, optic disc pit (ODP) maculopathy is another challenging condition where the lens capsule has shown utility. ODP maculopathy involves fluid seeping from a congenital or acquired pit in the optic disc into the macula, causing retinal detachment and schisis. Conventional treatments include vitrectomy with ILM peeling and gas, and sometimes placement of autologous tissues like a scleral plug or peripapillary laser to seal the pit. In 2019, Nakashizuka et al. reported using an anterior lens capsule flap as a physical plug for an acquired optic disc pit (an atypical case following glaucoma surgery) [63]. In that case, the patient’s lens capsule (harvested during phacoemulsification) was gently packed into the optic disc pit during vitrectomy. Gas tamponade was applied. Postoperatively, OCT confirmed complete closure of the disc pit and rapid resolution of subretinal fluid, with no recurrence at follow-up. The capsule remained stably wedged in the pit without causing inflammation. This novel approach essentially provided a biological cork to the leaking pit, analogous to how surgeons sometimes use a fragment of sclera or temporal fascia as a pit plug. The advantage of the lens capsule is that it is thin and flexible, it can conform to the pit cavity without exerting pressure on surrounding structures, and being transparent, it does not obscure the fundus view. Although data are limited to that report, the success suggests that capsular plugs could be considered in stubborn ODP maculopathy cases, especially acquired pits or large pits where ILM flaps are hard to secure [64]. It is a less traumatic alternative to a scleral autograft [65]. Further experience will determine whether this can become a standard technique; at the very least, it adds to the list of possible treatments when faced with persistent optic disc pit-related subretinal detachment.

In conclusion, the lens capsule has proven to be a versatile graft in the posterior segment for MH closure and optic disc pit sealing. Its use has extended the boundaries of what can be accomplished in eyes that previously had a poor prognosis due to the lack of ILMs or challenging disc defects. With meticulous surgical technique, capsular flaps achieve high anatomical closure rates and can significantly improve visual outcomes in these patients. Long-term follow-up and larger case series (and perhaps comparative studies against other techniques) will further clarify the benefits and any risks, but current evidence solidly supports the lens capsule’s role in retinal microsurgery.

Corneal Surface Repair and Bowman’s Layer Replacement

Corneal stroma and surface diseases have traditionally been managed with grafts like amniotic membranes or corneal transplants. The anterior lens capsule offers an intriguing alternative as a transparent patch graft for corneal surface restoration, particularly as a substitute for the Bowman’s layer or as a bandage in persistent epithelial defects.

Preclinical work in the early 2000s demonstrated that an isolated lens capsule can effectively replace Bowman’s layer in function. In Kozák’s 2003 study, rabbit eyes with refractory corneal ulcers had an allogeneic anterior lens capsule transplanted onto the denuded stroma after superficial keratectomy [10]. The transplanted capsule remained clear and allowed corneal epithelial cells to grow over it, resulting in healing of the epithelial defect. The cornea’s integrity was maintained, and the authors noted that the capsule “functioned as a Bowman’s membrane,” providing a smooth, adherent interface for the new epithelium. An electron microscopy analysis by Juhás et al. in 2004 confirmed that the lens capsule graft became well-integrated; hemidesmosomes formed between the new epithelium and the capsule, indicating good adhesion [34]. These findings suggested that the lens capsule could serve as a basement membrane substitute to support corneal epithelialization in cases where Bowman’s layer is damaged or absent.

In practice, the lens capsule is trimmed to size under the microscope and applied epithelial-side up, often secured using cyanoacrylate glue or fibrin sealant. Orientation and stromal contact are crucial for integration. To date, no randomized clinical trials have evaluated lens capsule transplantation in corneal surface disease. Future studies should assess not only epithelial healing but also visual quality, refractive changes, and graft clarity over time.

Clinically, the first human application of this concept was reported in 2025 by Kymionis and colleagues. They treated a patient with severe herpes simplex neurotrophic keratitis and a non-healing corneal ulcer by transplanting an allogeneic anterior lens capsule over the ulcer bed [43]. The donor capsule was placed much like an amniotic membrane graft, secured in position to cover the stromal defect. According to the case report, the lens capsule graft successfully promoted epithelial healing where other measures had failed. The cornea re-epithelialized over the capsule, restoring surface integrity and reducing stromal inflammation. Since the capsule is transparent, it provided an optically clear covering, and being a basement membrane, it likely supplied a favorable substrate with laminin and collagen IV for the patient’s epithelium to migrate on. This case established a proof of concept in a human clinical scenario, that an anterior lens capsule patch graft can rescue a refractory corneal ulcer by acting as a biological bandage contact lens or Bowman’s layer substitute.

Comparatively, amniotic membrane transplantation (AMT) has been the mainstay for such corneal surface reconstructions (Table 3). AMT modulates inflammation and angiogenesis and aids epithelial healing, but it eventually dissolves or is integrated, and it can become opaque and make postoperative evaluations difficult. The lens capsule, on the other hand, is an avascular, strong membrane that might stay longer at the transplant site. There is evidence that lens capsules can resist vascularization, since they contain collagen XVIII/endostatin, they might actively inhibit blood vessel in-growth. In rabbit experiments, capsules transplanted onto corneas remained avascular, whereas AMT might induce some superficial vascularization in a highly inflamed eye [10]. Also, the lens capsule’s tensile strength could help it act as a more robust tectonic support. For instance, in the case of a small corneal perforation or descemetocele, a lens capsule patch might physically plug the hole and add structural support similar to a corneal lamellar patch graft. It is speculated that lens capsule grafts could be used in corneal thinning or melts as an emergency patch, since the capsule is tough enough to hold sutures if needed.

**Table 3:** Comparative analysis of lens capsule versus amniotic membrane transplantation (AMT). AM: Amniotic membrane; AMT: Amniotic membrane transplantation.

Characteristic	Lens Capsule Transplantation	Amniotic Membrane Transplantation (AMT)
Tissue Origin	Autologous or donor human ocular tissue	Donor placental tissue
Composition	Type IV collagen, laminin, proteoglycans	Type IV & VII collagen, laminin, growth factors
Thickness	4-15 μm (Anterior Capsule); ~4 μm (Posterior Capsule)	20-50 μm (varies depending on preparation)
Transparency	Excellent; inherently transparent	Translucent; clarity decreases with integration
Mechanical Strength	High tensile strength, resilient, elastic	Moderate; thin AM tears easily, thicker AM is robust
Handling Ease	Easy (robust, curls slightly); holds sutures well	Moderate; thin AM is fragile, thicker AM is easier to handle
Availability and Banking	Harvested intraoperatively or from donor eyes; potential for banking	Established banking and widespread availability
Immunogenicity	Low to none (especially autologous)	Very low; immune-privileged tissue
Anti-inflammatory Properties	Theoretical; limited studies to date	Well-established anti-inflammatory cytokines
Antimicrobial Resistance	Theoretically high (resistant to enzymatic degradation); limited evidence	Moderate; indirect antimicrobial effects documented
Angiogenic/Anti-angiogenic Effects	Contains anti-angiogenic collagen XVIII (endostatin)	Contains anti-angiogenic and anti-scarring factors
Clinical Indications	Macular hole and optic pit repair, glaucoma bleb, corneal surface, basement membrane substitute	Ocular surface reconstruction, persistent epithelial defects, chemical burns, macular hole and optic pit repair
Regulatory and Ethical Issues	Minimal (autologous); moderate (donor)	Moderate (requires standardized donor screening)

A particularly fascinating emerging application is using the lens capsule in keratoconus surgery. Advanced keratoconus with severe thinning can sometimes be treated by Bowman layer (BL) transplantation, placing a donor BL into the cornea to stabilize shape (an alternative to full-thickness transplant). Donor BL tissue is scarce because it must be meticulously peeled from donor corneas. A recently published study (2024) introduced the intrastromal lens capsule graft for this purpose [8]. In that study, surgeons dissected a mid-stromal pocket in keratoconus corneas and inserted a rolled donor anterior lens capsule (from human eye bank eyes) into the pocket, unrolling it to span the central cornea. The capsule essentially took the place of a Bowman layer graft. At 1 month, treated corneas showed increased central thickness and some flattening of the cone, analogous to early outcomes of true BL grafts [8]. The capsule was visible on OCT as a thin line, but clinically the cornea remained clear. If further validated, this approach could provide a readily available alternative to donor corneas for keratoconus stabilization in selected cases, significantly broadening the therapeutic arsenal for corneal surgeons.

In the context of ocular surface tumors and scarring, the lens capsule also has potential. After surgical excision of lesions like conjunctival tumors or pterygia, or in conditions such as Stevens-Johnson syndrome with symblepharon, amniotic membrane is often used to reconstruct the surface. A lens capsule graft could similarly be laid over the sclera or cornea to facilitate epithelial regrowth and prevent adhesions. Its use in pterygium surgery (to cover bare sclera) has been suggested in theory, though no published series yet exists. Likewise, in recurrent erosion syndrome or epithelial basement membrane dystrophy (EBMD), where Bowman’s layer is dysfunctional, a lens capsule could be transplanted under the epithelium to create a new, smooth basement membrane (this is speculative but based on the same principle as in ulcers). One could envision a procedure where, after removing irregular epithelium and Bowman’s layer, a donor capsule is placed and the epithelium allowed to heal over it, potentially reducing recurrence of erosions.

The main considerations for corneal use are ensuring clarity and proper integration. In animal studies, lens capsules in the cornea have remained clear as long as the membrane is properly apposed and there is minimal interface opacity. If a capsule graft is too thick or multilayered, it could cause light scattering. Fortunately, anterior capsules are thin enough that a single layer is essentially transparent on the eye (similar to a healed LASIK flap). Another point is long-term behavior: does the capsule stay indefinitely or slowly get replaced by host collagen? Limited data from rabbits (up to ~6 weeks) showed the graft was still present. In a human eye, it’s conceivable the capsule could persist for years if not biodegraded. This could be beneficial (providing long-term support) but also means any future corneal procedure would encounter this layer.

In summary, for corneal surface repair, the lens capsule has demonstrated a capacity to act as a surrogate Bowman’s layer, promoting epithelial healing in refractory ulcers and possibly reinforcing the cornea in ectatic disorders. Early animal and human evidence is positive, and new surgical innovations (like intrastromal implantation for keratoconus) are expanding its role. As more cases are tried and reported, surgeons will better understand how to optimize this graft. It may not replace the amniotic membrane in all scenarios, but it offers an additional tool, particularly when autologous tissue is desired or when a sturdy, long-lasting membrane is needed on the cornea.

Tissue Engineering and Regeneration: Limbal Stem Cell Support, Endothelial Cells, RPE, IPE, and Optic Nerve

The search for ideal scaffolds for corneal cell transplantation has led researchers to natural membranes that mimic the cells’ native basement layers. The lens capsule, with its basement membrane composition and transparency, is well suited as a biological scaffold for corneal endothelial cells (CECs) and limbal stem cells.

Corneal endothelium: In endothelial keratoplasty (e.g., DMEK), donor Descemet’s membrane with endothelium is transplanted to replace diseased endothelium. Due to donor shortages, tissue-engineered grafts are being explored: culturing human CECs on a carrier substrate to create an implantable monolayer. The lens capsule has emerged as one of the most promising carriers for this purpose. Yoeruek’s 2009 study first indicated that human CECs can adhere and form tight monolayers on an isolated anterior lens capsule. Since then, advanced experiments have simulated the entire surgical process using capsule-based grafts [37]. Spinozzi et al. (2019) evaluated human anterior lens capsules (HALCs) as carriers for cultured porcine CEC sheets compared to other biomatrices [47]. They found that CECs on capsules achieved a confluent hexagonal monolayer with pump function proteins expressed, similar to those on natural Descemet’s membrane. Crucially, in a laboratory “insertion” test mimicking DMEK surgery, the capsule-cell constructs behaved very much like a native DMEK graft. The HALC with endothelium could be scrolled, inserted into an anterior chamber model, and unfurled without difficulty. It adhered well to the stromal bed after unfolding, whereas some synthetic scaffolds were either too floppy or too stiff. The study concluded that HALC was the most suitable carrier among those tested, allowing easy intraocular manipulation and good cell viability (over 95% cell viability on capsule vs. lower on some thicker collagen films) [47]. A follow-up study in 2020 went further, using human CECs on human lens capsules and achieving similar in vitro success [66]. Endothelial cell density and pump function on the capsule were comparable to controls, and the construct maintained transparency. These encouraging results suggest that clinical trials of lens capsule-based endothelial grafts for conditions like bullous keratopathy may soon be feasible. A patient’s own capsule (from cataract surgery) could even be used as the scaffold for expanding their endothelial cells in culture, then reinserted, a personalized endothelial graft without risk of rejection.

There are already indications that capsule-supported endothelial grafts can work in vivo. Nevertheless, a few hurdles remain: ensuring the capsule graft’s thickness does not impede vision (though an empty capsule is optically neutral once pressed against the posterior stroma), and ensuring firm long-term adhesion. Because the lens capsule lacks the anchoring glycoproteins found in natural Descemet’s membrane, it might be more prone to dislocation unless the surgical technique is perfected (perhaps via peripheral tucking or adhesive gels). These challenges can likely be addressed in animal models before human application. As of 2025, no registered human trials have evaluated HALC-supported CEC transplantation in vivo, though preclinical handling and cell viability studies are promising.

Limbal epithelial stem cells:The lens capsule has been successfully used as a substrate to grow limbal stem cell cultures for ocular surface reconstruction. This approach aims to treat limbal stem cell deficiency (LSCD) by growing new corneal epithelium from a small limbal biopsy on a carrier, then transplanting that sheet onto the patient’s cornea. Conventionally, human amniotic membrane is used as the culture substrate and transplant carrier for limbal cells. Galal et al. (2007) offered the lens capsule as an autologous scaffold alternative. In their study, limbal biopsies from patients were cultured on the patients’ own anterior lens capsules (harvested during cataract surgery) [11]. After two weeks, they reported robust epithelial outgrowth with cell density, morphology, and viability matching those grown on plastic or amniotic controls. Over 95% of cells remained viable and stratified into a multilayer epithelium, with formation of cell junctions (desmosomes) between layers. The authors concluded that the human lens capsule is a viable scaffold for ex vivo expansion of limbal epithelial cells, with the advantage of being autologous (eliminating immunologic concerns). They envisioned a scenario in which, during cataract surgery, one could collect the capsule and a tiny limbal biopsy, expand the cells on the capsule in the lab, and then place that capsule, with new epithelium, onto the patient’s scarred cornea. This technique, if realized, would avoid the use of xenogeneic feeder cells or animal products, particularly if human serum is used in culture (as Albert et al. did in 2012) [35,36].

Albert et al. refined this method by using an animal product-free culture system, employing human serum as the only supplement and the lens capsule as the substrate [36]. They demonstrated that outgrowth from limbal explants on the capsule occurred as early as 24 hours, with >97% cell viability and expression of key limbal stem cell markers (p63α, ABCG2). The expanded cells retained a less differentiated phenotype, positive for stem cell markers and negative for CK3/CK12 (corneal differentiation markers), indicating that the capsule microenvironment did not push them into premature differentiation. Colony-forming efficiency was also confirmed, meaning the stem cells remained potent [36]. This work provides a strong foundation for lens capsule-supported limbal epithelial transplantation in the future. Essentially, it could allow completely autologous cultured epithelial grafts (the scaffold and the cells are both autologous), which is highly desirable for avoiding rejection and ethical issues.

The clinical translation of limbal cell-on-capsule grafts is still pending, but conceptually, it is straightforward. For clinical application, Good Manufacturing Practice (GMP)-compliant labs must be used to prepare cell-seeded capsules, utilizing xeno-free media and conducting pathogen screening for both donor capsules and patient cells. During surgery, the cell-laden capsule could be draped onto the cornea and secured with fibrin glue or a single suture at the limbus. The capsule would serve as a temporary basement membrane beneath the new epithelium. Over time, it might integrate into the healing anterior stroma or remain as a thin interface layer. Because it is essentially the same type of membrane as Bowman’s, it could potentially become incorporated and even indistinguishable as healing progresses, providing a long-term foothold for the transplanted epithelium.

In both endothelial and limbal cell contexts, the biocompatibility of the lens capsule with human ocular cells is a recurring theme. These cells behave on the capsule much as they do on their native substrates: endothelial cells pump fluid and form cell junctions, while limbal epithelial cells stratify and maintain stemness. The capsule provides natural cues (via its ECM proteins) that synthetic scaffolds often cannot. Moreover, being naturally thin and transparent, it doesn’t impede light, critical for corneal applications.

In summary, the lens capsule has proven to be an excellent support membrane for cell culture and transplantation in ophthalmology. For corneal endothelial cell therapy, it offers a natural, Descemet’s-like carrier that can be easily handled during surgery and may help alleviate donor shortages by enabling lab-grown grafts [47]. For limbal stem cell therapy, it provides an autologous, xeno-free scaffold to grow healthy corneal epithelium for patients with ocular surface failure [36]. These applications are on the cusp of clinical reality, with in vivo trials likely forthcoming. The success in these areas underscores a paradigm: the lens capsule can serve as a universal basement membrane substitute for various ocular cells, leveraging nature’s own design to support regeneration.

RPE and IPE regeneration: One additional application to note is the support of RPE or IPE. As mentioned earlier, Hartmann et al. (1999) showed that RPE and iris pigment cells attach and grow on lens capsules in vitro [32]. They even attempted grafting such constructs into rabbit models. In the realm of stem cell therapy for AMD, where RPE cells derived from stem cells are transplanted on scaffolds, some researchers have proposed using the human lens capsule as the implantation scaffold due to its Bruch’s-like properties. Another study by Nicolini et al. (2000), referenced by Kiilgaard, likely conducted similar experiments, reinforcing the concept [49]. Microcontact printing techniques have also been studied on human lens capsules to guide RPE growth into desired patterns. One group printed grid patterns of adhesive molecules on capsules to encourage organized RPE monolayers, demonstrating that the capsule can function as a tissue engineering platform [67]. The printed capsule supported RPE maturation and could be handled for transplantation. This approach may be applicable to future regenerative therapies for retinal diseases. The subretinal space, specifically the interface between the neurosensory retina and the RPE/Bruch’s membrane, is another area where the lens capsule has been tested as a substitute support structure. In conditions like age-related macular degeneration (AMD) or retinal degenerations, the idea of transplanting healthy RPE cells or retinal sheets is often limited by the absence of a healthy Bruch’s membrane. A stable subretinal scaffold could help such transplanted cells survive and organize. The lens capsule, with its Bruch’s-like permeability and structural proteins, has been explored in this context.

As discussed, Kiilgaard et al. (2002) conducted a landmark study in pigs, implanting allogenic anterior lens capsules into the subretinal space [33]. The surgical method involved a vitrectomy and retinotomy to insert the capsule beneath the retina. They observed that when the native Bruch’s membrane was not intentionally damaged, host RPE cells began migrating onto the capsule and formed a confluent monolayer on its surface within 11 days. The newly formed RPE layer appeared normal and healthy. This key result indicates that the capsule can function as an artificial Bruch’s membrane, allowing RPE to attach and thrive. The capsule did not induce an inflammatory reaction on its own; ocular tolerance was good. However, the study also revealed a cautionary finding: if Bruch’s membrane was purposely ruptured (simulating a situation like AMD, where Bruch’s is diseased or absent), the capsule alone could not completely prevent undesirable growth, choroidal fibrovascular tissue tended to invade and proliferate onto the capsule from underneath. In other words, the lens capsule provides a surface for RPE above, but it is not a complete barrier to cells and vessels from below if Bruch’s is absent, at least not in that model. This suggests that in cases of macular degeneration with active neovascularization, a capsule graft might require adjunctive measures (e.g., an anti-VEGF environment or edge sealing) to stop choroidal vessels from crossing over.

So how might this translate clinically? One scenario is the repair of RPE defects after CNV removal. In some surgical cases of AMD (historically, submacular surgery to remove CNV membranes), patients were left with an RPE-denuded area under the fovea, leading to poor vision. Surgeons attempted to transplant autologous RPE from the periphery or even move the retina (macular translocation). A lens capsule graft could theoretically be placed in such an RPE-free zone to act as a new Bruch’s, followed by seeding it with RPE cells (either autologous peripheral RPE or stem-cell-derived RPE). The capsule would provide the substrate for those cells to adhere and organize, which a scarred Bruch’s could not. The permeability of the capsule is advantageous: it would allow nutrients from the choroid to diffuse to the RPE and photoreceptors, similar to native Bruch’s membrane. Indeed, a study measured the molecular weight cutoff of the lens capsule and found it comparable to human Bruch’s, supporting this use-case [68].

So far, clinical use in humans for subretinal support has been very limited. There have been experimental human trials using artificial substrates (like polyester membranes) to implant RPE in AMD, but those had issues such as rigidity and long-term biocompatibility. The lens capsule could be a more biocompatible alternative. Given the success in pigs, it’s plausible someone will attempt it either in a clinical trial for geographic atrophy (placing iPSC-derived RPE on a lens capsule under the macula) or in a desperate case of RPE loss [31].

One interesting twist is the use of IPE. Some researchers have tried transplanting IPE cells to the subretinal space to replace lost RPE (since IPE are embryologically similar). Hartmann’s study indicated lens capsules can also support IPE sheets [32]. Although IPE transplants have largely fallen out of favor due to limited functional success, the capsule could serve as a common denominator as a scaffold for any cell type we want to put under the retina.

Overall, the lens capsule shows promise as a temporary or permanent Bruch’s membrane substitute, especially to aid RPE transplantation. Its strengths lie in biocompatibility and its ability to promote cell monolayer formation. Its weaknesses may lie in not being completely impermeable to aggressive fibrosis or vessels from below, meaning it might work best in relatively controlled environments (e.g., dry AMD or cleaned subretinal space) rather than in actively neovascular ones.

Another speculative subretinal use is in retinal prosthesis or gene therapy delivery. A lens capsule could theoretically serve as a scaffold to deliver sheets of gene-corrected RPE or even act as a support for a layer of stem-cell-derived photoreceptors placed subretinally, keeping them aligned. These are forward-looking ideas.

In conclusion, while not yet in routine clinical practice, the lens capsule’s role in the subretinal space has been validated in preclinical models. It can support RPE attachment and survival, effectively standing in for Bruch’s membrane in experimental retinal surgery. The challenge lies in preventing underlying scar tissue in pathological settings, but with adjunctive therapies, the capsule could become part of a future RPE transplantation toolkit for macular diseases. It exemplifies how a structure from the anterior segment can be repurposed to solve problems in the posterior segment, a true cross-regional application of a natural scaffold.

Neuro-regenerative contexts:The lens capsule has been identified as a permissive substrate for axonal growth, as demonstrated in rat optic tract injury models, suggesting its potential to address optic nerve injuries [69,70]. Possible mechanisms include ECM-integrin interactions promoting neurite outgrowth, structural guidance from collagen fibrils, and immunologic quiescence reducing glial scar formation. These properties make lens capsules promising for promoting neural regeneration, beyond traditional ophthalmology, highlighting their innovative potential in neuro-regenerative contexts. While whole globe transplantation is a highly complex and non-standard procedure, integrating lens capsules into this process could theoretically enhance its neuro-regenerative potential by providing a scaffold for axonal growth within the transplanted eye. Recent advancements in whole-globe transplantation, such as the first combined face and whole-eye transplant performed in 2023, demonstrate the feasibility of such procedures and may pave the way for future innovations in vision restoration (NYU Langone Health, 2024) [70,71]. However, the use of lens capsules in this context remains speculative and requires further research to validate efficacy. To date, studies of axonal growth over lens capsule substrates have been limited to rodent models, with no clinical application; the concept remains entirely preclinical [69,70].

Glaucoma Bleb Reinforcement

Trabeculectomy and other bleb-forming glaucoma surgeries often face the issue of postoperative fibrosis, which can cause the filtering bleb to fail. Surgeons routinely apply antimetabolites like mitomycin-C (MMC) or 5-FU to suppress scarring. The anterior lens capsule has emerged as a novel mechanical and biological spacer that can be placed under the scleral flap to maintain bleb patency and potentially reduce fibrosis, effectively reinforcing the bleb from within.

The idea behind using a lens capsule in trabeculectomy is two-fold: (1) it physically props open the scleral flap, creating a micro-reservoir and preventing the flap from sealing completely, and (2) it provides a barrier that might modulate healing responses (similar to how an MMC-soaked sponge works, but here the barrier remains in place). Additionally, because the capsule is acellular (after prep) and collagenous, it might discourage fibroblasts from contracting the area, and it has been speculated to have anti-fibrotic or anti-angiogenic properties (perhaps due to factors like collagen XVIII in it)​ [1].

Clinically, the use of autologous lens capsules in combined phacoemulsification-trabeculectomy was first reported by Lu et al. in 2009 [12]. They compared eyes that received a piece of the patient’s anterior capsule under the scleral flap to those that did not. While their study was relatively small, it suggested improved bleb function in the capsule group. A larger and more robust study by Das et al. in 2016 provided high-quality evidence. In that prospective randomized trial (88 eyes), the surgical technique for the experimental group involved performing phacoemulsification, saving the anterior capsule disc, then conducting a standard trabeculectomy with the trimmed capsule disc placed underneath the scleral flap before suturing [41]. No MMC or 5-FU was used, relying solely on the capsule for bleb modulation. The outcomes at 3 months clearly favored the capsule group: Complete success (IOP ≤21 without meds) was 93.2% vs 70.5% in controls and the mean IOP was ~11 mmHg in the capsule group vs 17 mmHg in controls. Moreover, bleb grading showed more diffuse, functional blebs (Grades I-II) in the capsule group, whereas the control group more often had high, encapsulated blebs (Grade IV)​. This suggests that the capsule helped form a healthier filtering bleb structure. Complication rates were low and similar in both groups; importantly, there were no instances of bleb-related infections or persistent hypotony attributable to the capsule.

Surgeons who have used the technique describe that the capsule is placed over the sclerostomy and under the flap, like an internal bandage. It tends to flatten out and cover the scleral opening. When the scleral flap is sutured over it, the flap doesn’t lie completely flat against the sclera; the capsule maintains a small gap that facilitates aqueous percolation into the subconjunctival space. Postoperatively, the capsule likely also acts as a baffle, spreading the aqueous flow more evenly. Histologically (in rabbit models or hypothetically in humans), one might expect less connective tissue contraction around the area, since the capsule is inert and perhaps separates the healing edges.

An interesting observation from the 2016 study was that even without antimetabolites [41], the outcomes with capsule augmentation were on par with what one might expect using MMC. This raises the possibility that in certain patients, for instance, those at risk for MMC complications (thin conjunctiva, young patients, ocular surface disease) or in resource-limited settings, using the patient’s own lens capsule could circumvent the need for anti-fibrotic drugs. The capsule is free, autologous, and avoids the well-known risks of MMC (e.g., scleral thinning, avascular blebs, late infection, etc.).

There are some practical considerations: This technique is obviously only available to phakic patients who are undergoing cataract removal or who are willing to undergo lens removal at the time of trabeculectomy. In younger patients without cataracts, one wouldn’t sacrifice the natural lens just to obtain the capsule, so its use is naturally geared toward combined cataract-glaucoma surgeries. Fortunately, many glaucoma patients do have cataracts (or lens changes) by the time surgery is needed, so it can align well. In pseudophakic patients, one could consider using a donor capsule (though that has not yet been reported for trabeculectomy, it could be feasible via eye bank tissue).

How does a capsule-augmented bleb fare long-term? The published follow-ups are short (3-6 months). We would want to know whether the IOP advantage persists at 1 year, 2 years, etc. There’s a possibility that scarring could still catch up later, although one could hypothesize that the initial healing phase is the most critical, and by placing a capsule, a favorable bleb formation pattern might be set and sustained. Even if some scarring occurs, the bleb formed might be more diffuse and functional. Anecdotally, some surgeons have reported seeing the capsule in the bleb on gonioscopy or anterior segment OCT postoperatively as a translucent sheet. It likely remains in place for a long time (the capsule doesn’t dissolve readily). If a reoperation is needed (e.g., bleb revision), encountering the capsule is an interesting scenario, one could potentially lift or adjust it.

In any case, the concept of a “biologic spacer” in glaucoma surgery is now established by these studies. The lens capsule joins other materials like collagen matrices and amniotic membranes that have been used to modulate bleb healing. For example, the amniotic membrane has been placed under scleral flaps in some studies to improve bleb outcomes, but amnion can become vascularized or be absorbed. The lens capsule appears to resist such issues and integrates well. It is also less immunogenic than a dermal collagen implant.

In summary, the use of the lens capsule in glaucoma surgery is an elegant repurposing of tissue to address one of the fundamental challenges in trabeculectomy, scarring. Clinical evidence (including a randomized study) indicates that an autologous capsular graft under the scleral flap can significantly enhance the success of the surgery without additional complications [41]. This could herald a shift in how we approach combined cataract-glaucoma operations, potentially reducing reliance on anti-fibrotic drugs. As more surgeons become aware of the technique, we may see it adopted, especially in cases where MMC is contraindicated, or when performing phaco-trab in one go (achieving cataract removal, IOP control, and using the byproduct of the former to improve the latter). The potential use of lens capsules as spacers around microshunts, such as the PRESERFLO MicroShunt, remains to be explored, as their biocompatibility and structural properties could theoretically enhance the stability and positioning of these devices in glaucoma surgery.

Capsular Bag Transplantation

The technique of capsular bag transplantation involves harvesting a donor capsular bag, often with an IOL, and implanting it into the recipient's eye to address conditions such as aphakia and aniridia. Kondratenko and Yakimov pioneered this method, demonstrating its feasibility in restoring the eye's anatomical integrity [72].

In their approach, an open-sky capsulorhexis is performed on a donor eye, followed by hydrodissection and removal of the lens material. The capsule is then stained with trypan blue to enhance visualization, and the zonules are severed using scissors. Silicone-covered forceps are used to transfer the capsule. Two delivery techniques have been introduced:

Pull technique: utilizes vacuum through a silicone tube to pull the capsule into the recipient's eye.

Push technique:involves loading the capsule into a cut phaco silicone sleeve and injecting it into the eye, akin to the method used in Descemet Membrane Endothelial Keratoplasty (DMEK) with an IOL cartridge.

Once inside the recipient’s eye, the bag is filled with an ophthalmic viscosurgical device (OVD) and held in place using iris hooks. A capsular tension ring (CTR) is manually implanted into the bag to restore its shape, and capsular tension segments (CTS) are used to anchor the bag to the sclera with sutures, ensuring stable long-term fixation. Finally, a monofocal acrylic one-piece IOL is implanted into the reconstituted bag.

Building upon this foundation, Abbas Khoja et al. reported successful capsular bag transplantation in cases of post-traumatic aniridia with aphakia and uveitis-glaucoma-hyphema (UGH) syndrome [59]. Their approach involved preserving the donor capsular bag through freezing prior to implantation. A pars plana vitrectomy was performed in each case, and the in-the-bag IOL complex was fixated using the cow-hitch suture technique. The transplanted capsular bags remained stable and well-positioned without any signs of rejection, effectively restoring anatomical compartmentalization and preventing mechanical trauma from the IOL to adjacent ocular structures.

In addition to donor-derived solutions, artificial capsular devices have been developed to mimic the natural lens capsule's function, providing support for IOL implantation in the absence of sufficient capsular support.

VaMa (Vatavuk and Marić) artificial IOL capsule: This experimental device is crafted from polydimethylsiloxane (PDMS) silicone elastomer and features a universal design with a 10 mm outer diameter. The VaMa capsule is engineered to accommodate various types of IOLs, facilitating in-the-bag implantation even in patients lacking sufficient capsular support. Its design includes multiple miniature holes along the peripheral edge for anchoring, allowing for stable fixation within the eye. This artificial capsule aims to enable proper positioning and potential future exchange of IOLs, addressing challenges associated with aphakia and damaged native capsules [73].

Gemini refractive capsule: Developed by Omega Ophthalmics, the Gemini Refractive Capsule is a three-dimensional implant inserted into the capsular bag through a small incision, designed to maintain the bag’s openness. The Gemini capsule can house a proprietary, exchangeable optic and provides space for drug delivery, biometric sensors, and additional lens technologies. Its structure aims to reduce the risk of capsular fibrosis and opacity, offering a stable platform for IOLs and potentially enhancing surgical outcomes [74].

These advancements underscore the evolving landscape of ophthalmic surgery, where both biological and synthetic solutions are being explored to address complex anterior segment challenges. The integration of such innovative techniques and devices holds promise for enhancing surgical outcomes and expanding treatment options for patients with compromised capsular support.

Table 4 provides a summary of some of the interspecies and inter-tissue applications we have discussed, illustrating the breadth of scenarios where lens capsules have been, or could be, utilized. This is a prime example of the lens capsule’s versatility: from the posterior pole of the retina to under the conjunctiva in a bleb, it finds use across the eye.

**Table 4:** Examples of lens capsule applications across species and tissues. As Table 4 illustrates, the lens capsule has demonstrated utility in multiple compartments of the eye and even across different species. Its cross-species performance (e.g., porcine to rabbit, porcine to pig, human to rabbit) has generally been favorable, thanks to its properly prepared acellular nature. ALC: Anterior Lens Capsule; PL: Posterior Lens Capsule; RPE: Retinal Pigment Epithelium; MH: Macular Hole.

Donor/Host Context	Application	Outcome
Rabbit ALC to rabbit cornea (allograft) [34]	Chronic corneal ulcer (basement membrane repair)	Complete re-epithelialization with normal basement membrane and hemidesmosomes; no rejection​.
Human ALC (autograft) to cornea [11]	Limbal stem cell expansion (ex vivo)	Successful growth of limbal epithelium on capsule, 95% cell viability, confluent layer​.
Porcine ALC to rabbit cornea (xenograft) [8]	Intrastromal Bowman’s layer substitute (keratoconus model)	Graft remained clear and integrated; cornea maintained shape and thickness, no inflammation​.
Human ALC from FLACS donors to cornea [42]	Biological drape for corneal perforation	Favorable healing reported; procedural details on storage, sterilization, and donor status not disclosed; reproducibility uncertain
Human donor capsular bag [59,72]	Management of aphakia, zonular deficiencies,and aniridia	Improved IOL stability and visual rehabilitation; require further long-term evaluation
Human ALC (autograft) to retina [75]	Macular hole patch (refractory MH)	High closure rate of large macular holes (up to 100% in series); capsule visible on OCT aiding retinal bridging​.
Human PLC (autograft) to retina [75]	Macular hole patch (when ALC unavailable)	Achieved closure in ~50% of cases; more difficult handling, outcome inferior to ALC.
Porcine ALC to pig subretinal space (allograft) [33]	RPE support in RPE-transplant model (Bruch’s replacement)	Host RPE formed monolayer on capsule with normal morphology; capsule tolerated without inflammation.
Porcine ALC to pig subretinal (with seeded RPE) [49]	RPE transplantation scaffold (AMD model)	Cultured RPE attached and survived on pALC; no immune reaction at 2 wks, though graft curling noted.
Human ALC (autograft) to optic nerve head [63]	Optic disc pit maculopathy (patching the pit)	Successful pit closure, resolution of macular fluid; ALC remained in place with no complications​.
Human ALC (autograft) in trabeculectomy (phacotrab) [41]	Bleb support to reduce scarring (glaucoma)	Lower IOP and higher success vs control at 3 months; capsule under flap maintained filtration, no adverse events​.

Critical appraisal of current studies

While the above paints an optimistic view, it’s crucial to critically analyze the literature:

Small Sample Sizes and Case Nature

Many reports are case series or even single cases (e.g., optic pit, neurotrophic keratitis). This is expected in early-stage innovation but means results could be skewed by publication bias, successes are reported, failures less so. For example, Peng et al. had 10 eyes, Chen & Yang had 20, and Yepez had 2, underpowered to detect rare complications or generalize findings across diverse patient populations. Future larger studies or clinical trials are needed.

Lack of Controls

Almost none of these studies included a control group. Ideally, one might compare autologous capsule vs. ILM flap vs. no treatment in refractory MHs, or capsule vs. amniotic membrane (AM) in corneal ulcers. The absence of direct comparisons forces reliance on historical success rates. Peng’s 90% closure looks impressive against the ~50-70% reported in literature for other methods in similar large holes, but this remains an indirect comparison [39].

Short Follow-Up

Visual and anatomical outcomes are typically reported at 3-12 months. The long-term stability of these grafts is not fully known. Will an autologous capsule in the macula remain in place 5-10 years later? Some suggest it might, due to lack of rejection, but could it contract or migrate? Will an allograft capsule in the cornea eventually dissolve, or conversely, trigger a late immune response? Kiilgaard’s pig study lasted 49 days, and Juhás’s rabbit model extended to 3 months, still short relative to the human lifespan [33].

Orientation and Technique Nuances

Some failures or complications (like gliosis over a closed MH) might stem from subtle technical errors, perhaps the capsule was placed upside down (i.e., with the stromal side up rather than the basement membrane side), altering Müller glia interactions. Few major papers detail orientation; only Peng’s method mentions insertion under the retina, implying the basement membrane side likely faced the retina. A deeper understanding of correct orientation and technique will improve with experience.

Biological Interaction Uncertainties

While the lens capsule is relatively inert, its behavior in new environments remains unclear. In MH surgeries, do Müller cells migrate onto it and stop, or do they over-proliferate? There is some indication that autologous blood may promote glial bridging, aiding closure but possibly encouraging scar tissue. In the cornea, does a lens capsule encourage deposition of native basement membrane proteins over time, becoming integrated into host tissue? Rabbit data showing hemidesmosome formation is promising. Histological analysis of human samples (e.g., from failed grafts or enucleated eyes) would be invaluable to evaluate in vivo remodeling over months or years.

Handling and Standardization

The surgical community lacks standardized protocols for lens capsule handling. Currently, different groups use varied methods. Chen & Yang didn’t use blood [38], while Peng did [39]. Some stained the capsule with dyes like BBG or ICG; others did not. There's no consensus on whether to trim the capsule slightly larger than the hole (as Peng did), or leave it untrimmed (as Chen likely did). Should intraoperative OCT be used to confirm positioning? Some authors noted limitations due to lack of iOCT. Surgical guidelines are needed for reproducible, safe application.

Surgical familiarity with handling transparent capsular flaps is limited. Training modules, wet lab simulations, and step-by-step technical guides will be essential to overcome the procedural learning curve.

We also note that publication bias may play a role: lens capsule transplants that didn’t work out might not be written up. Surgeons with suboptimal outcomes may quietly abandon the technique. Thus, while the existing literature is promising, it may also be selectively optimistic.

Speculative applications of lens capsule transplantation

The lens capsule’s unique biomechanical integrity, collagen IV-rich matrix, and immune-privileged status have inspired a range of experimental and theoretical applications across ocular and systemic contexts. While many current uses are clinical or in preclinical stages, the following speculative applications highlight emerging directions in regenerative medicine, device integration, and ocular surface reconstruction.

Ocular Surface Reconstruction

In cases of pterygium recurrence, conjunctival tumors, chemical burns, Stevens-Johnson Syndrome (SJS), or graft-versus-host disease (GVHD), the lens capsule may serve as a transparent biological membrane for ocular surface healing. The lens capsule, thin, pliable, and avascular, could theoretically be used to reconstruct the tarsal conjunctiva or to line symblepharon release beds, providing a biological scaffold to support epithelial regrowth. Its dense, collagenous nature resists degradation by collagenases, making it particularly suited for conditions involving stromal melts or corneal ulceration. Unlike amniotic membrane, the lens capsule may offer longer persistence and transparency without the risk of vascularization. Notably, anecdotal evidence suggests that capsules remain intact even in eyes with active endophthalmitis, hinting at resistance to inflammation and microbial degradation. Although direct studies confirming resistance to macrophages, neutrophils, fungi, and bacteria are currently lacking, the capsule’s structural resilience and collagen IV matrix make it a strong candidate for surface stabilization in hostile environments. Another potential application is in cases of band keratopathy or superficial scars: after scraping calcium or performing PTK, a capsule layer might be added to protect the stroma and provide a smooth interface for epithelium, potentially improving healing and vision.

Biomechanical Enhancement for Keratoconus

The application of UV radiation to lens capsule tissue may induce crosslinking and increase its rigidity, theoretically enhancing its suitability for implantation in keratoconic corneas as a biomechanical stabilizer. This would parallel the concept of UV crosslinking in corneal collagen and may be combined with intrastromal implantation techniques currently explored for Bowman layer transplantation. Though untested in clinical trials, this approach offers a potential alternative for biomechanical reinforcement in ectatic diseases.

Capsular Wrapping of Glaucoma Microshunts

The capsule sac may be adapted as a biologic wrapper around subconjunctival microshunts (e.g., PRESERFLO MicroShunt) to modulate healing and prevent fibrosis. Its structural flexibility and immunological inertness could reduce fibroblast adhesion and minimize bleb encapsulation. The capsule’s ability to conform tightly around devices while preserving aqueous outflow dynamics makes this an appealing hypothesis warranting further investigation.

Barrier Grafting in Complex Aphakia

In eyes with aphakia and concurrent silicone oil tamponade, the absence of a native capsule allows oil to migrate anteriorly, risking corneal damage and glaucoma. A grafted capsule, sutured to the sulcus, iris remnants, or anterior chamber angle, may serve as a biological barrier, mimicking the natural posterior capsule. This could also be applied in DSEK or DMEK procedures in aphakic eyes, where a capsule could act as a plug to cover the pupil, support graft positioning, and prevent posterior migration of the graft or gas. The capsule could later be dissected with YAG lasers. Scleral fixation of the capsule in the absence of zonular support remains hypothetical but conceptually analogous to artificial capsule devices.

Drug Delivery Reservoirs

A decellularized lens capsule sac (Figure 1E) could be filled with pharmacologic agents and implanted in the subconjunctival space or anterior chamber. Its semi-permeable nature would permit sustained drug release, similar to other slow-release depots [76]. For instance, anti-VEGF agents, corticosteroids, or antibiotics could be encapsulated in a sealed or sutured capsule pouch to deliver localized therapy in chronic uveitis, macular degeneration, or post-surgical inflammation. Experimental validation is needed, but the concept aligns with the broader field of biologically integrated drug delivery systems.

Cell Encapsulation for Tissue Engineering

The lens capsule has been proposed as a scaffold for cell therapy due to its immune privilege and ability to support epithelial adhesion. A particularly novel concept involves encapsulating insulin-producing beta cells within the capsule sac (Figure 1E), creating a biological barrier that allows nutrient diffusion while shielding the cells from immune attack [77]. Lens material from young donor eyes could be extracted, and the remaining empty capsule resealed to form a protective chamber. This could represent a step toward bioengineered pancreatic implants without systemic immunosuppression. Similarly, RPE, corneal endothelial cells, or stem-cell-derived tissues could be embedded within lens capsule constructs for subretinal or anterior segment applications.

In summary, these speculative directions expand the landscape of lens capsule applications from current retinal, corneal, and glaucoma uses into device integration, immunoprotected cell therapy, and ocular surface reconstruction. Each idea remains hypothetical, requiring rigorous biomechanical, immunological, and surgical validation. However, the capsule’s inherent properties continue to inspire cross-disciplinary innovation, positioning it as an adaptable platform for future ophthalmic and systemic regenerative therapies.

Challenges and future perspectives

While the lens capsule has demonstrated remarkable versatility and safety in the eye, several challenges must be addressed before it can be widely adopted and its full potential realized. Additionally, there are opportunities to enhance and expand its use through further research.

Standardization and Supply

A major limitation is the availability of suitably prepared lens capsules. Unlike amniotic membranes, which are banked and distributed by eye/tissue banks, there is not yet an established system for collecting and storing lens capsules on a large scale. Most reported uses have been autologous during the patient’s own surgery, or small-scale allografts arranged ad hoc. Developing a standardized protocol for eye banks to harvest anterior lens capsules from donor eyes (or even from the thousands of cataract surgeries done daily) could create a ready supply of tissue. This requires coordination with surgeons and ensuring sterile collection. If achieved, surgeons could order capsules (cryopreserved or lyophilized) much as they do an amniotic membrane or donor cornea. It will be important to validate preservation methods, e.g., confirming that a frozen-thawed capsule retains tensile strength and biocompatibility. Researchers might compare cryopreserved versus fresh capsules in animal models for any differences in outcome.

Surgical Innovation

Surgeons will likely invent new delivery techniques for the lens capsule. A preloaded injector for intraocular use, e.g., a cartridge that holds a rolled capsule for MH repair, could make it easier to insert through a small cannula without losing it. Finer instruments to manipulate capsules may be needed; since capsule flaps are tougher than ILM, perhaps micro-forceps can be designed with a slightly broader gripping surface to hold without tearing. Glue and capsule combos, as seen in corneal surgery (e.g., tissue adhesives like fibrin glue), may pair well with capsules. We might see kits that include a glue and membrane for various uses. For suturing capsules as patch grafts on sclera or conjunctiva, fine sutures (10-0 nylon or Prolene) or new suturing devices could help tack them on without tearing, perhaps with a small ring of thicker material at the edge to anchor stitches.

Interspecies Considerations

The success of pig-to-rabbit and pig-to-pig experiments suggests that xenografts might be feasible in humans (with proper cleaning). Porcine lens capsules are appealing because pig eyes are abundant byproducts of the food industry, and their size is similar to human eyes. There may be minor compositional differences; one study noted differences in cell adherence between porcine and bovine capsules​ [32], possibly due to species-specific matrix nuances or freshness. If xenografts were used clinically, thorough decellularization would be non-negotiable, and perhaps even antigen-reduction treatments (e.g., removing species-specific epitopes) could be added. That said, collagen IV and laminin are highly conserved proteins, so an acellular xenograft is unlikely to be rejected.

Mechanical Properties and Optimization

The lens capsule is elastic, it stretches and recoils with accommodation in the lens. In a new location (cornea or retina), this elasticity might cause it to behave in unexpected ways (like curling, as noted in subretinal placement​). Researchers might explore ways to modulate the stiffness of the capsule for specific uses. For instance, gentle cross-linking (using UV-A and riboflavin) could stiffen an ALC if needed for corneal use to better mimic Bowman’s rigidity. Conversely, partial digestion or thinning might make an ALC more flexible for subretinal use. One intriguing possibility is engineering composite grafts, e.g., bonding an ALC to a thin synthetic mesh or a softer biomaterial to tailor its properties. However, such complexity might not be necessary, given the capsule’s innate suitability in many cases.

Size and Scaling Issues

The small size of the capsule (especially autologous capsulorhexis buttons, ~5 mm) is a constraint for covering larger defects. This can be partly mitigated by creative surgical techniques, such as using multiple capsules or cutting and unfolding a whole donor capsular bag to cover a broader area (like the "candy wrap" approach; Figure 1C). This raises the idea of patchworking multiple capsules or using a capsule in combination with other grafts (perhaps an ALC centrally for optical clarity, with an amniotic membrane peripherally). One future idea is to bioengineer larger capsule-like membranes by growing lens epithelial cells in vitro to secrete a bigger capsule or layering capsules. However, that is complex. In the interim, targeting uses that match the capsule’s size, like MHs, small corneal ulcers, and focal patches, will be most practical. For widespread ocular surface reconstruction (which might require 2-3 cm² of membrane), the capsule might serve as a supplement rather than the main graft due to area limitations.

Biomechanical Longevity

We need more data on how lens capsule grafts fare in the long term. Do they remain intact and functional years after transplantation? For example, in an anterior capsule patch in the cornea, will it degrade or calcify after many years? Collagen IV membranes are generally stable, but changes might occur in certain conditions, such as prolonged exposure to tear film. Follow-up of patients who have received these grafts (like those in MH or trabeculectomy studies) beyond the initial few months will be valuable. If a graft fails or contracts, understanding the mode of failure will guide improvements.

Immune and Histologic Response

While the lens capsule itself is acellular and should not provoke a strong immune rejection, any allograft in living tissue can elicit some response over time. It will be important to observe whether implanted capsules cause any chronic inflammation or if the body eventually treats them as native tissue. The early evidence is reassuring (e.g., no inflammation in pig subretinal implants​ [33]; no graft rejections in allograft capsules for optic pit​ [63]), but systematic studies could confirm this. Histology from animal models in which capsules were implanted for months could show how host cells interact with them, e.g., do host fibroblasts infiltrate the capsule, or do they form a layer over it? Understanding the integration process will help predict longevity.

Safety and Complications

So far, reported complications are minimal. As seen in the rare case of MH gliosis, if a capsule is not properly decellularized, residual lens epithelial cells or other cells can proliferate and form unwanted membranes [53]. This underscores the need for meticulous preparation. In future use, especially allografts, standardized decellularization (e.g., a quick distilled water rinse or enzyme treatment) should be protocol. Some scenarios might benefit from pre-treating the capsule with anti-scarring agents, for example, soaking the capsule in MMC briefly before use under a trabeculectomy flap might further reduce fibrosis. However, this could also weaken the capsule; such trade-offs need exploration. There is also a theoretical risk of capsule opacification: lens capsules can accumulate calcium or pigment under certain conditions (e.g., partial fibrosis in cataract surgery if lens epithelium remains). Ensuring no lens cells remain should avoid any fibrotic opacification. Still, more research is needed on the cellular interactions at the graft interface.

No evidence that transplanted capsules calcify spontaneously is available, but long-term observation is needed. If a capsule in the cornea were to calcify, it could become opaque. Another risk is infection, any implanted material can be a nidus, but since the capsule is avascular, infection would likely only occur if the surface was compromised (e.g., an epithelial defect on a corneal capsule graft could let bacteria in). Standard infection prophylaxis (antibiotic drops post-op) has been used in all cases, just as one would in any corneal procedure. 

Does the capsule merely sit there as a passive matrix, or do host cells infiltrate it over time? Some histology from corneal cases indicates host fibroblasts can migrate into the periphery of an ALC graft, but central parts remain acellular and clear. Long-term, the capsule could potentially become covered by a thin fibrous layer, which might or might not affect transparency. Encouragingly, in a chronic rabbit cornea study, even at 3 months the epithelium was well-adherent, and the interface was smooth with the graft [34], implying minimal scar tissue formed on top of the capsule.

Attachment and Fixation Methods

Depending on location, one challenge is how to secure the capsule in the desired position during healing. In MHs, surface tension and tamponade or blood clot suffice. In corneas or sclera, sutures or fibrin glue have been used for analogous grafts. The capsule can be delicate to suture (risk of tearing), but it actually holds sutures reasonably well due to its collagen framework. Surgeons will need to adapt techniques, perhaps using a “capsule clip” or ring for certain placements (imagine a tiny ring that can hold a capsule in the pupil for the silicone oil barrier idea). For conjunctival or surface uses, tissue glue might be ideal to avoid needle holes. The development of specialized instruments or devices to handle the ultra-thin capsule might also ease some procedures (e.g., a preloaded capsule injector or intraocular capsule manipulator).

Regulatory and Acceptance Hurdles

Introducing a new surgical material, even autologous, will require careful evaluation. If using autologous tissue, there’s little barrier to adoption besides surgeon training. For allogeneic or xenografts, tissue handling standards akin to those for donor corneas or amnion would apply. Regulatory bodies may eventually require eye bank-processed capsules to meet sterility and safety criteria. Given that the capsule is not a drug or cellular product, it might be regulated similarly to an acellular tissue matrix (like commercial pericardium or decellularized corneal patches), which are generally classified as HCT/P (human cell/tissue products) with minimal manipulation, thus, possibly not requiring full FDA premarket approval if done within a tissue bank context for homologous use. However, these details are jurisdiction-dependent.

Clinical trials for new indications (e.g., keratoconus BL substitute) will help generate data. Surgeons must also climb a learning curve to become comfortable handling the capsule, which is a different skill from handling synthetic materials. Sharing of techniques and outcomes in the literature will accelerate adoption. It may also be challenging to convince surgeons to change practice when existing solutions (like ILM for MHs, or MMC for trabeculectomy) work reasonably well. Thus, demonstrating clear advantages or unique niches (e.g., cases where other methods cannot be used) will be key to acceptance.

Enhancement via Bioengineering

Looking forward, one can imagine enhancing the lens capsule for specific tasks. For example, embedding growth factors or extracellular matrix cues into the capsule to promote healing, maybe coating it with fibronectin for faster epithelialization on the cornea, or impregnating it with antiproliferative drugs for glaucoma. Because the capsule is somewhat porous, drugs could potentially be loaded for slow release. Another idea: combining the capsule with cells, like seeding it with corneal endothelial cells (which is being done in labs) and using it as a living graft, or seeding with stem cells for the ocular surface. These bioengineered constructs will need validation, but they represent a synergy of scaffold and cell therapy.

Cross-Disciplinary Use

Outside of pure ophthalmology, the lens capsule might find roles in adjacent fields, for instance, as a scaffold for culturing tissue in vitro (e.g., researchers have used lens capsules to grow multilayer cell cultures to test drugs). Interdisciplinary collaboration between corneal surgeons, retinal surgeons, tissue engineers, and immunologists will enrich the research on lens capsule applications. Could lens capsules be used outside the eye? Perhaps in ENT (e.g., patching tympanic membrane perforations), it’s speculative, but their size is similar to small perforations. Or in neurosurgery to patch the dura (though pericardium is common and thicker).

In terms of future perspectives, The trajectory of lens capsule use in ophthalmology appears to be on an upswing. What was once an overlooked remnant is now being reconsidered as a multi-purpose graft and scaffold. We anticipate several developments in the coming years:

Controlled trials and expanded case series for each application*: *How does the lens capsule compare to the current gold standard? This question will guide clinical adoption. For example, in ocular surface reconstruction, amniotic membrane is the gold standard, so future trials could compare AM vs. ALC grafts for recurrent erosions or acute burns. If ALC grafts yield faster epithelialization or clearer corneas, that would be a win. In MHs, initial data suggest ALC flaps achieve similar or better closure rates compared to amniotic membrane inserts (which have been tried but sometimes cause inflammation). In keratoconus, a formal comparison between donor Bowman’s layer grafts and ALC grafts would be enlightening; the latter avoids the use of scarce donor tissue, but we must ensure it offers equivalent structural support.

New case reports pushing into the speculative areas: For instance, a first report of using a capsule in pterygium surgery or in a scleral melt, if successful, could spark further use. Tissue bank programs starting to save capsules, even a pilot program where surgeons can request a preserved capsule for tricky cases, would be a big step.

Product development: Perhaps a commercial entity might create a pre-packaged sterile lens capsule (possibly from an animal source) for surgical use, similar to how pericardium patches are sold. If regulatory hurdles are cleared, this could bring a “Capsule as On-Demand Patch Kit” to any OR without needing a cataract operation. It’s conceivable that ophthalmic surgeons might keep a few sterile capsules in their clinic to use for emergency repairs (like a small perforation). The development of a commercial Lens Capsule Patch product could facilitate this. Ensuring consistent quality (size, thickness, clarity) will be key. Capsules from younger donor lenses might be ideal for certain uses (younger lenses have slightly thinner ALC, ~10 μm [15], vs. older donors ~15 μm, which might be too stiff for fine retinal work). So donor selection could matter.

Integration with cell therapy: Given the extensive research in stem cell and regenerative therapy in ophthalmology, the lens capsule could become a natural scaffold of choice for implanting lab-grown tissues (whether endothelium, epithelium, or retinal cells). If, say, an iPSC-RPE therapy for AMD needed a scaffold, a decellularized human lens capsule might be an option to carry the cells into the subretinal space (some studies are indeed exploring this). Thus, the capsule might play a role in cutting-edge therapies beyond traditional surgery.

Capsule modifications: Impregnating capsules with growth factors or drugs, for instance, loading an ALC with epidermal growth factor (EGF) to accelerate epithelialization when used as a patch, or anti-VEGF in a subretinal capsule for AMD, offering structural support plus biochemical therapy.

The diverse applications reviewed above establish the lens capsule as a multi-talented graft material with parallels to the amniotic membrane and other biologics, yet with unique advantages (and some challenges). In facing these challenges, the field can draw lessons from how other tissues entered practice (e.g., how amniotic membrane overcame initial skepticism through sustained outcomes and easy availability). The lens capsule’s strength lies in its origin, it is part of the eye, so it inherently behaves well in the eye. Harnessing that is a matter of refining techniques and proving benefits.

In conclusion, the lens capsule has transitioned from being surgical waste to a resource of great interest. Its versatility in form and function, from acting as a substrate for cell growth to serving as a structural patch, opens many possibilities. The current evidence, spanning the ocular surface to the retina, consistently highlights its biocompatibility, transparency, and ease of use once proper preparation protocols are followed. We have only begun to scratch the surface of what could be done with this material. As technology advances (e.g., more precise lasers for harvest, better tissue engineering techniques), we may integrate lens capsules into complex ocular bioimplants or even prosthetic devices. For practicing clinicians, the near-term takeaway is: think outside the lens. Next time you remove a cataract, consider that the capsule you’re peeling might save someone’s cornea or macula. With further research and clinical experience, the lens capsule could become a standard tool in the ophthalmologist’s armamentarium, embodying the concept of recycling biological tissue for therapeutic gain. The overlooked capsule is finally getting the attention it deserves, and future directions point to an expanding role in ophthalmic surgery, potentially improving outcomes in some of the most challenging conditions we face.

## Conclusions

This review has highlighted how the lens capsule’s unique combination of transparency, biomechanical strength, and biocompatibility enables it to serve as a multifunctional scaffold for closing macular holes, patching corneas, supporting cultured cells, or reinforcing a glaucoma bleb. Clinical reports and studies to date demonstrate that lens capsule grafts can achieve outcomes comparable to or surpassing traditional methods in select cases, all while utilizing autologous or donor tissue that integrates naturally into the eye.

From a retinal surgeon’s perspective, the capsule offers a new solution for otherwise intractable problems like chronic macular holes and optic disc pit maculopathy, providing high anatomical closure rates where ILM or other tissues fall short. For corneal specialists, the capsule opens possibilities for biologic bandage contact lenses in non-healing ulcers and even innovative procedures such as intrastromal keratoconus grafts, leveraging its role as an analogue to Bowman’s layer. Glaucoma surgeons see in the capsule a potential replacement for anti-fibrotic chemicals, a way to naturally improve bleb function and safety by implanting the patient’s own membrane under the scleral flap. The lens capsule is a truly multipurpose ocular biomaterial, able to interface with various cell types (neurosensory retina, RPE, corneal epithelium, endothelium, fibroblasts) and encourage desired healing patterns. Key to these successes are the capsule’s intrinsic properties: it is thin yet handleable, biologically compatible, and integrates with surrounding tissues without inducing scarring or rejection.

Furthermore, the differences between the thicker anterior capsule and the ultra-thin posterior capsule have been leveraged to tailor their use, the robust anterior lens capsule excels as a structural patch (as in macular holes and corneal grafts), while the delicate posterior lens capsule more closely mimics membranes like the ILM or Descemet’s, though with handling challenges. Comparative studies across species confirm that basement membrane composition is conserved and that xenografts can be tolerated after decellularization, pointing to the possibility of animal-derived capsule grafts as a resource. We have also seen that innovations in harvesting and preservation are bringing capsule grafts closer to routine clinical reality. Simple methods such as water, salt, and alcohol soaks can produce sterile, cell-free capsules suitable for transplantation.

Looking ahead, the future directions for the lens capsule are expansive. As tissue banks refine preservation techniques, we can anticipate readily available allogenic lens capsule grafts, which will greatly facilitate research and clinical adoption. Further studies are likely to solidify protocols, for instance, the optimal method to decellularize and sterilize capsules without compromising their integrity or the best suturing techniques for different indications. Integration with regenerative medicine approaches is especially exciting: we may soon see clinical trials where stem-cell-derived corneal endothelium on a lens capsule is transplanted into patients with corneal edema, or where a sheet of iPSC-derived RPE on a capsule is placed under a degenerating macula. In such scenarios, the lens capsule is not just a passive scaffold but an active enabler of cell therapy, providing a biomimetic environment that supports cell survival and function (the lens capsule).

It is foreseeable that eye banks may soon store human lens capsules much like they do amniotic membranes, making them readily available in operating rooms. The ongoing advancements in ophthalmic surgery, such as femtosecond laser technology or Zepto PPC technology, synergize with the concept, enabling precise procurement of capsular tissue. As more surgeons report their experiences and refine techniques, a standardized approach to lens capsule use will crystallize.

Of course, prudent evaluation of long-term outcomes and any unforeseen effects is necessary. The ophthalmic community will need to gather evidence on how these capsular grafts perform over the years, and whether any complications emerge with larger sample sizes. Thus far, the absence of immune reactions or significant complications in published cases is reassuring, attesting to the capsule’s immune privilege and safety profile. With growing clinician familiarity and patient awareness, lens capsule grafting could transition from innovative case reports to mainstream practice for specific indications. It may not replace established therapies outright, but rather complement them, for example, reserved for cases where standard treatments are not possible or have failed.

In conclusion, the overlooked lens capsule is being reinvented as a valuable resource in ophthalmic surgery. It exemplifies the principle of 'using nature’s design': a basement membrane that has evolved to support lens cells can be repurposed to support healing in other ocular tissues. The current applications underscore the capsule’s role as a biological patch, plug, and scaffold, while future applications may well expand its utility to even broader horizons, from ocular surface reconstruction to retinal tissue engineering. As knowledge and experience continue to accumulate, ophthalmologists may soon routinely reach for the lens capsule, a once-forgotten tissue, as a go-to solution in challenging cases. Embracing this naturally derived, biocompatible material aligns with the trend toward regenerative and autologous therapies, ultimately improving patient outcomes while minimizing foreign implants. In the story of surgical innovation, the humble lens capsule is poised to go from castaway to cornerstone, truly exemplifying overlooked versatility now coming to light.
